# Biodegradable and Dual‐Responsive Polypeptide‐Shelled Cyclodextrin‐Containers for Intracellular Delivery of Membrane‐Impermeable Cargo

**DOI:** 10.1002/advs.202100694

**Published:** 2021-07-18

**Authors:** Sergej Kudruk, Sharafudheen Pottanam Chali, Anna Livia Linard Matos, Cole Bourque, Clara Dunker, Christos Gatsogiannis, Bart Jan Ravoo, Volker Gerke

**Affiliations:** ^1^ Institute of Medical Biochemistry Center for Molecular Biology of Inflammation University of Muenster Von‐Esmarch‐Str. 56 Münster 48149 Germany; ^2^ Center for Soft Nanoscience and Organic Chemistry Institute University of Muenster Busso Peus Straße 10 Münster 48149 Germany; ^3^ Center for Soft Nanoscience and Institute of Medical Physics and Biophysics University of Muenster Busso Peus Straße 10 Münster 48149 Germany; ^4^ Max Planck Institute of Molecular Physiology Otto‐Hahn‐Straße 11 Dortmund 44227 Germany

**Keywords:** biodegradable, cyclodextrin, dual‐responsive, intracellular delivery, polypeptides

## Abstract

The transport of membrane impermeable compounds into cells is a prerequisite for the efficient cellular delivery of hydrophilic and amphiphilic compounds and drugs. Transport into the cell's cytosolic compartment should ideally be controllable and it should involve biologically compatible and degradable vehicles. Addressing these challenges, nanocontainers based on cyclodextrin amphiphiles that are stabilized by a biodegradable peptide shell are developed and their potential to deliver fluorescently labeled cargo into human cells is analyzed. Host–guest mediated self‐assembly of a thiol‐containing short peptide or a cystamine‐cross‐linked polypeptide shell on cyclodextrin vesicles produce short peptide‐shelled (SPSV_ss_) or polypeptide‐shelled vesicles (PPSV_ss_), respectively, with redox‐responsive and biodegradable features. Whereas SPSV_ss_ are permeable and less stable, PPSV_ss_ effectively encapsulate cargo and show a strictly regulated release of membrane impermeable cargo triggered by either reducing conditions or peptidase treatment. Live cell experiments reveal that the novel PPSV_SS_ are readily internalized by primary human endothelial cells (human umbilical vein endothelial cells) and cervical cancer cells and that the reductive microenvironment of the cells’ endosomes trigger release of the hydrophilic cargo into the cytosol. Thus, PPSV_SS_ represent a highly efficient, biodegradable, and tunable system for overcoming the plasma membrane as a natural barrier for membrane‐impermeable cargo.

## Graphical abstract

Transport of membrane impermeable compounds into the cell's cytosolic compartment should ideally be controllable and involve biologically compatible and degradable vehicles. Addressing these challenges, nanocontainers based on cyclodextrin amphiphiles that are stabilized by a biodegradable peptide shell are developed and their potential to deliver fluorescently labeled or biological active cargo into human cells is shown.

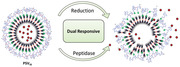

## 1 Introduction

Nanocontainers have been used in the past as vehicles for the targeted release of active substances,^[^ ^1^, ^2^, ^3^ ^]^ as nanoreactors,^[^ ^4^, ^5^ ^]^ as delivery systems for vaccines^[^ ^6^, ^7^ ^]^ and as intracellular delivery tools for amphiphilic cargo such as fluorescently labeled lipids.^[^ ^8^ ^]^ Because of their excellent biocompatibility, lipid vesicles, which consist of a lipid bilayer enclosing an aqueous lumen, have been extensively studied and have also been used in clinical applications.^[^ ^9^, ^10^, ^11^, ^12^ ^]^ Other approaches introduced polymer‐stabilized nanocontainers^[^ ^13^ ^]^ that typically exploit the structural advantages of supramolecular self‐organization and provide precise control of the nanosize morphology as well as modular modification options.^[^ ^14^ ^]^

Self‐organizing principles were employed by, for example, Hotz et al.,^[^ ^15^ ^]^ who used the limited space of the bilayer membrane of vesicles for a cross‐linking polymerization of hydrophobic monomers to form polymer nanocapsules. An alternative approach established polymer‐stabilized cyclodextrin nanocontainers to encapsulate hydrophilic or amphiphilic cargo.^[^ ^8^, ^16^, ^17^ ^]^ In view of biomedical and cell biological applications, it is advantageous for nanocontainers to be able to release an enclosed cargo in a controlled manner, possibly even in a specific cellular microenvironment such as endosomes or lysosomes.^[^ ^2^ ^]^ This has been addressed in recent years, in particular by exploiting endogenous cellular conditions such as low lysosomal pH value or intracellular redox potential as release stimuli.^[^ ^18^, ^19^, ^20^, ^21^, ^22^, ^23^ ^]^ Redox‐sensitive nanocontainers, for example, rely on the strongly reductive microenvironment in cells, which is achieved by high glutathione concentrations (1–10 mm)^[^ ^24^ ^]^ and in endosomal compartments by the activity of gamma interferon‐induced lysosomal thiol reductase (GILT).^[^ ^25^ ^]^ However, redox‐sensitive nanocontainers employed so far utilized disulfide groups introduced in an organic polymer shell representing a non‐biological structure.

Therefore, we developed a nanocontainer strategy based on a peptide shell that guarantees high biocompatibility. Combined with a redox‐sensitive cyclodextrin core, this strategy led to the generation of dual responsive containers, carrying a polypeptide shell of tunable thickness. The containers demonstrated remarkable biodegradability as well as biocompatibility and enabled the controlled intracellular delivery of hydrophilic cargo into various types of cells which occurred via endocytosis of the containers and a redox/protease‐dependent cargo release from endosomes.

**Figure 1:**
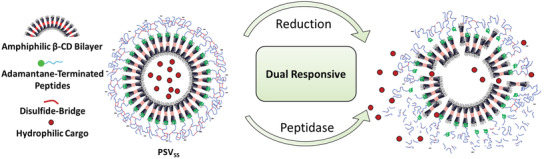
Schematic representation of the self‐assembly of amphiphilic *β*‐CD and adamantane‐terminated peptides followed by disulfide formation and cystamine‐cross‐linking resulting in peptide‐shelled vesicles PSV_SS_. Reduction of the disulfide bonds and proteolytic cleavage are expected to dissociate the polymer shell into smaller molecular units (amino acids or short peptides) thereby revealing the permeable cyclodextrin core.

The rationale of our dual responsive approach is outlined in **Figure** **1**. Hydrophilic cargo and *β*‐cyclodextrin amphiphiles (*β*‐CD) were co‐assembled into liposome‐like cyclodextrin vesicle (CDV) templates and decorated by host–guest chemistry with a reductively cleavable polypeptide shell. Encapsulation and delivery efficiency of these biodegradable nanocontainers were analyzed for different subtypes generated by changing the length of the peptide sequence in the shell. For this purpose, cyclodextrin vesicles were either decorated with thiol‐containing short‐peptides (*n* = 6–9) to provide short peptide‐shelled vesicles (SPSV_SS_) cross‐linkable by oxidation, or decorated with glutamic acid‐containing polypeptides (*n* = 100 ± 5) to provide polypeptide‐shelled vesicles (PPSV_SS_) which were cross‐linked using cystamine.

## 2 Results and Discussion

**Figure 2:**
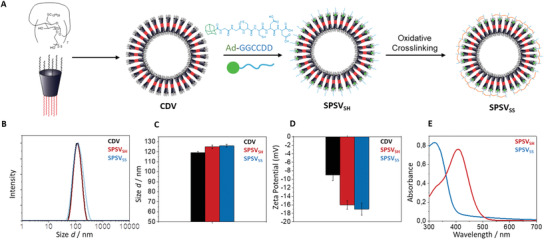
Preparation and biophysical characterization of SPSV_SS_. A) Self‐assembly of amphiphilic *β*‐CD into vesicles, attachment of adamantane‐terminated short peptides such as Ad‐GGCCDD to form SPSV_SH_ and cross‐link of the cysteine moieties by oxidation yields the short peptide cyclodextrin containers, SPSV_SS_. B) Intensity‐weighted size‐distribution of the different intermediates and the final SPSV_SS_ product determined by DLS. CONTIN‐algorithm for polydisperse samples was used for the analysis. C) Bar graph comparison of the size distribution between CDV, SPSV_SH_, and SPSV_SS_. D) Zeta potential of CDV, SPSV_SH_, and SPSV_SS_. E) Absorbance spectra of Ellman's test^[^ ^27^ ^]^ performed with SPSV_SH_ and SPSV_SS_, respectively, showing the different absorbance peaks of the reduced and oxidized form of the Ellman's reagent. The absorbance maximum at 410 nm indicates the presence of accessible thiols.

Redox‐sensitive short‐peptide cyclodextrin vesicles (SPSV_SS_) were assembled as depicted in **Figure** **2**A. Briefly, amphiphilic *β*‐CD was synthesized as described,^[^ ^26^ ^]^ hydrated and then extruded to yield CDV with an average hydrodynamic diameter of *d*_h_ ≈ 119 nm and a *ζ* potential of *ζ* ≈ −9 mV that remained stable over several days (Figure 2B–D). In the next step, CDV were decorated with varying adamantane‐terminated short peptide sequences via host–guest interaction. The synthesis of thiol‐containing Ad‐GGCCDD, Ad‐TEG‐GGCCDD, Ad‐GGGCCCDDD, and non‐thiol containing Ad‐GGGDDD is described in the Supporting Information. The formation of homogenous particles, herein referred to as short peptide‐shelled cyclodextrin vesicles (SPSV_SS_), was assessed by dynamic light scattering (DLS) and *ζ*‐potential measurements. In the case of Ad‐GGCCDD containing SPSV_SS_, this revealed an increase of the hydrodynamic diameter by 7 nm and a decrease in the *ζ*‐potential from *ζ* = −9 to −17 mV due to deprotonated carboxylic acid moieties (Figure 2B–D). The stability of the amino acid shell was increased by oxidative cross‐linking, yielding a disulfide bridge between the cysteine units, and the absence of free thiol groups in the oxidated product was proven by reaction with Ellman's reagent 5,5′‐dithiobis‐(2‐nitrobenzoic acid), which produces an absorbance peak of the cleaved di‐anionic product 2‐nitro‐5‐thiobenzoate only with free thiols^[^ ^27^, ^28^ ^]^ (Figure 2E).

A further characterization of Ad‐TEG‐GGCCDD, Ad‐GGGCCCDDD, and Ad‐GGGDDDD decorated SPSV_SS_ is given in Figures S1–S5, Supporting Information. These data reveal that the size distribution after the host–guest attachment of adamantane‐terminated peptides to the CDV increased by *d*_h_ ≈ 5–8 nm and that the *ζ*‐potential decreased to a value ranging from *ζ* = −14 to −7 mV with lowest *ζ*‐potentials observed for the highest number of terminal aspartic acid units. However, when SPSV_SS_ with an encapsulated fluorescent cargo such as pyranine or carboxyfluorescein were prepared, they showed a substantial leakage (i.e., rapid loss of fluorescent cargo) rendering this approach inappropriate for cellular delivery (Figure S5D, Supporting Information).

**Figure 3:**
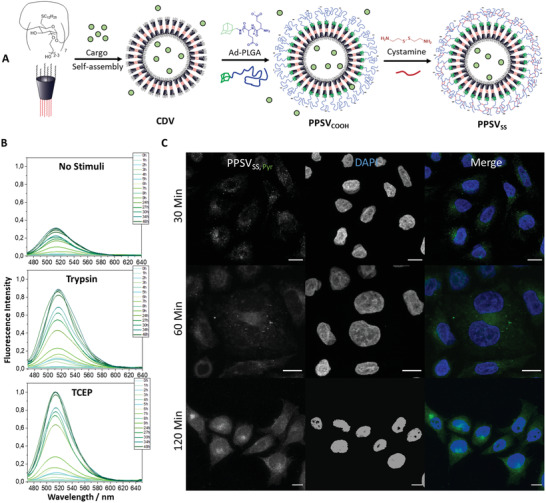
Dual responsiveness and cellular uptake of PPSV_SS_. A) Scheme depicting the preparation of PPSV_SS_. Self‐assembly of amphiphilic *β*‐CD into vesicles (CDV) is followed by attachment of adamantane‐terminated polypeptides Ad‐(Glu)_100±5_ to form PPSV_COOH_ which are stabilized by cross‐linking of the carboxylic acid moieties with cystamine to form the polypeptide‐shelled cyclodextrin containers, PPSV_SS_. B) Fluorescence spectra of the time‐dependent pyranine release from PPSV_SS_. Nanocontainers were either left untreated (no stimuli), or incubated with trypsin or TCEP. PPSV_SS_ were kept in dialysis containers and the pyranine fluorescence in the surrounding dialysis medium was recorded as a means to determine the release of cargo. C) Confocal images of HeLa cells incubated with pyranine‐loaded PPSV_SS_ and fixed after 30, 60, and 120 min. Note the efficient uptake and punctate as well as general cytosolic appearance of the fluorescent pyranine. With prolonged time puncta become less abundant and cytosolic fluorescence with some perinuclear enrichment is observed (green channel represents pyranine; blue channel represents DAPI chosen to stain nuclei). Scale bar = 10 µm.

To reduce this spontaneous leakage from the nanocontainers, we increased the shell thickness by using longer peptides and thus generated polypeptide shelled cyclodextrin vesicles (PPSV_SS_) (**Figure** **3**A). Synthesis of adamantane‐terminated poly‐glutamic acid by *N*‐carboxyanhydride polymerization, self‐assembly of redox‐responsive nanocontainers, and cystamine‐mediated cross‐linking that establishes a disulfide bridge between the polypeptides were carried out as described^[^ ^29^ ^]^ and specified in the Supporting Information (Figures S6–S8, Supporting Information). *ζ*‐potential measurements and DLS revealed a successful formation of the nanocontainers (Figure S8A–C, Supporting Information). Importantly, DLS showed a monodisperse distribution, and neither disruption of the vesicles nor aggregation by intervesicle cross‐linking were observed. An increase in average size from 126 nm in CDV to 134 nm in PPSV_SS_ indicated the presence of polypeptides on the surface of the nanocontainers. Another verification of successful host–guest interaction was the decrease in *ζ*‐potential from −9 to −15 mV due to the incorporation of the negative charge of glutamic acid. After cross‐linking and converting the acid group to an amide‐bond the *ζ*‐potential increased from −15 to −14 mV.^[^ ^8^, ^17^ ^]^ By quantifying the reaction of fluorescein‐5‐maleimide with free thiol groups in cross‐linked PPSV_SS_ as compared to PPSV_SS_ fully reduced with tris(2‐carboxyethyl)phosphine‐hydrochloride (TCEP), the cross‐linking efficiency was determined to be ≈9% (Figure S8D, Supporting Information). Cross‐linked PPSV_SS_ showed an average hydrodynamic diameter of *d*_h_ ≈ 145 ± 10 nm and appeared as mostly circular, hollow objects in cryo electron microscopy (cryoEM) images (Figure S9, Supporting Information). While Volta phase plate cryoEM revealed a layer thickness of around 5 nm corresponding to a CDV bilayer, the polypeptide shell could not be discerned because of its low electron density. The data obtained in the DLS and cryoEM measurements are fully consistent and in line with previous reports on the size and morphology of CDVs and polymer‐shelled CDVs.^[^ ^8^, ^17^, ^30^ ^]^

Cellular uptake of nanosized vehicles can occur through various mechanisms such as membrane fusion,^[^ ^31^ ^]^ caveolin‐mediated endocytosis,^[^ ^32^, ^33^ ^]^ clathrin‐mediated endocytosis,^[^ ^33^, ^34^ ^]^ micropinocytosis,^[^ ^35^ ^]^ and phagocytosis.^[^ ^36^ ^]^ Therefore, we analyzed next whether and how PPSV_SS_ are internalized by cultivated human cells after verifying that PPSV_ss_ are basically non‐toxic to cultured cells at the concentrations employed here (Figure S10, Supporting Information). To visualize the behavior of PPSV_SS_ in live cells, the nanocontainers were derivatized with an amine‐functionalized fluorophore, Dy633, which was attached during the cross‐linking process by covalent linkage to carboxylic acid moieties of the poly‐glutamic acid shell. Figure S11, Supporting Information, shows that the Dy633‐labeled PPSV_SS_ were readily internalized into cervical cancer cells (HeLa) cells with the label appearing in punctate structures that were identified as endosomes by simultaneous uptake of fluorescein‐conjugated dextran (M_n_ ≈ 10 kDa). Similar results were obtained for primary human umbilical vein endothelial cell (HUVEC) indicating an efficient endocytic uptake of PPSV_SS_ into human cells from different origin (Figure S11, Supporting Information).

Endosomes and lysosomes exhibit a reductive environment due to the presence of reduced glutathione, GILT,^[^ ^37^ ^]^ protein disulfide‐isomerases like^[^ ^38^ ^]^ thioredoxin‐like protein TXNL1,^[^ ^39^ ^]^ and antioxidant proteins such as peroxiredoxin 4.^[^ ^40^ ^]^ Moreover, endosomes/lysosomes harbor as one class of acid hydrolases peptidases that are activated at the low endosomal pH, for example, signal peptide peptidase,^[^ ^41^ ^]^ papain‐like peptidases, chymotrypsin‐like cysteine 3c‐like peptidases,^[^ ^42^ ^]^ and cathepsins.^[^ ^43^ ^]^ In order to exploit this reductive and proteolytic endosomal microenvironment, we next tested whether PPSV_SS_, as anticipated in their design, respond to redox changes and the activity of proteolytic enzymes. Therefore, we employed nanocontainers loaded with pyranine, decorated by adamantane‐terminated polyglutamic acid (Ad‐PLG_90_ or Ad‐PLG_105_) and cross‐linked with cystamine (Figure 3A). In these pyranine‐loaded PPSVss (PPSV_SS, Pyr_), the dye loading content, which is the ratio of the weight of dye inside the PPSV_SS, Pyr_ to the weight of the nanocontainer without cargo calculated using the dye absorption spectra, was determined for Ad‐PLG_90_ to be 2.9% and for Ad‐PLG_105_ to be 3.5% (Figure S8E, Supporting Information). The regulated release of pyranine was then examined followed by the treatment with trypsin as a peptidase or TCEP as the reducing agent. Figure 3B shows that efficient release of dye into the medium surrounding the PPSV_SS_ and was observed in both cases. Under these conditions, TCEP treatment resulted in an almost complete release of pyranine, and trypsin incubation led to an ≈90% release efficiency as compared to less than 30% of fluorescence release from the non‐treated PPSV_SS_. A moderate, non‐stimulated leakage was also observed for other cyclodextrin‐based nanocontainers (polymer‐shelled CDVs) where it ranged from 20% to 40% either for encapsulated pyranine or carboxyfluorescein after 48 h.^[^ ^17^ ^]^ Most likely, this background leakage is due to a moderate degree of cross‐linking of the polypeptide (or polymer) shell (9% according to Figure S8, Supporting Information). Moreover, repulsive forces of the negatively charged polypeptide (or polymer) chain could result in increased leakiness of the shell. Nonetheless our findings so far suggest that encapsulated cargo will undergo enhanced release from PPSV_SS_ inside the reductive and proteolytic environment of endosomes.

Given the efficient endocytosis of PPSV_SS_ in mammalian cells (Figure S11, Supporting Information) and their redox and protease response (Figure S12, Supporting Information), we next analyzed the intracellular fate of the pyranine‐loaded nanocontainers (PPSV_SS, Pyr_) following uptake into HeLa cells. Figure 3C shows that 30 min after incubation of cells with the PPSV_SS, Pyr_, the membrane‐impermeable pyranine label appeared in structures most likely resembling endosomes and was also present faintly throughout the cytosol. This cytosolic appearance becomes even more pronounced at longer incubation time (60 min and longer) and quantification of the efficiency of PPSV_SS, Pyr_ uptake and pyranine release into the cytosol at 4 h post addition revealed successful cytosolic dye delivery in 47.8 ± 1.1% of HUVEC and 40.4 ± 3.1% of the HeLa cells (Figure S13, Supporting Information). Thus, in contrast to the labeled polypeptide shell that remained in endosomes for an extended period of time, the small hydrophilic cargo pyranine is released into the cytosol. Most likely, this occurs because the cleaved polypeptide‐envelope of the PPSV_SS_ behaves like polyanionic substances, which are known to destabilize endosomes by affecting the membrane potential thereby enabling the passive diffusion of endosomal contents into the cell's interior.^[^ ^44^, ^45^ ^]^ This behavior is fundamentally different from that of viral fusion proteins^[^ ^46^ ^]^ or cell‐penetrating peptides, for example, HIV‐derived HGP peptide,^[^ ^47^ ^]^ TAT‐fusion peptide,^[^ ^48^ ^]^ or papillomavirus L240 peptide.^[^ ^49^ ^]^

**Figure 4:**
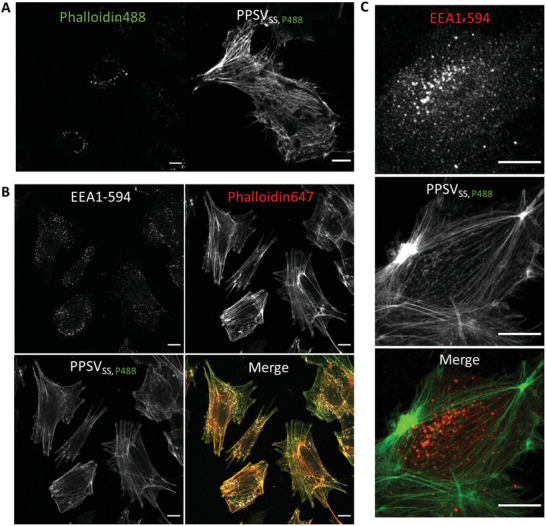
Intracellular delivery of hydrophilic cargo encapsulated in PPSV_SS_. A) Live HeLa cells were incubated with iFluor phalloidin488 alone used as control (left) or with PPSV_SS, P488_ for 120 min and then analyzed by confocal microscopy. Note that the membrane‐impermeable iFluor phalloidin488 is only transported into the cellular cytosol to stain filamentous structures when encapsulated in the polypeptide‐shelled containers PPSV_SS_ (right panel). The few punctate structures positive for iFluor phalloidin488 seen in the left panel most likely represent aggregated material on top of the cells. B) Confocal images of HeLa cells treated with PPSV_SS, P488_ for 120 min, fixed and permeabilized. Cells were costained with a mouse antibody directed against the early endosomal protein EEA1 and an Alexa Fluor 594 labeled secondary anti‐mouse antibody (AF‐594) as well as with iFluor phalloidin647. No presence of iFluor phalloidin488 in early endosomal structures positive for EEA1 could be observed at this time point of incubation, whereas an almost complete colocalization is observed with phalloidin647 given after the permeabilization. C) Primary human endothelial cells (HUVEC) were incubated with PPSV_SS, P488_ for 120 min, then fixed, permeabilized, and treated with EEA1 mouse antibodies (EEA1) followed by Alexa Fluor 594 labeled secondary anti‐mouse antibody (AF‐594). Scale bar = 10 µm.

To show that the biodegradable and redox/protease‐responsive nanocontainers can deliver hydrophilic drugs into the cellular cytosol, we next loaded PPSV_SS_ with the fungal toxin phalloidin which was fluorescently labeled with an iFluor488 dye (PPSV_SS, P488_). Phalloidin is a bicyclic heptapeptide that selectively binds to and stabilizes actin filaments and, among other things, is widely used to label the actin cytoskeleton.^[^ ^50^ ^]^ However, so far its use is restricted to fixed and permeabilized cells as phalloidin cannot pass through cell membranes.^[^ ^51^ ^]^ When HeLa cells were treated with the dual‐responsive PPSV_SS, P488_ containers, efficient release of iFluor phalloidin488 into the cytosol could be observed as evidenced by the pronounced staining of cytosolic filamentous structures representing F‐actin (**Figure** **4**A). This release occurred in a time‐dependent manner and appears complete after 2 h. Importantly, the imaging revealed that the real‐time behavior of F‐actin can be recorded in live cells. As expected, increased cell poisoning was observed with prolonged incubation, confirming the progressive endosomal release of iFluor phalloidin488 (Figure S12C, Supporting Information). Next, we analyzed whether the iFluor phalloidin488 released from endocytosed PPSV_SS, P488_ containers accessed all cytosolic actin filaments, thereby permitting a full visualization of cytosolic F‐actin in live cells. Therefore, cells were first treated with PPSV_SS, P488_ containers to allow cytosolic delivery of iFluor phalloidin488, and then fixed, permeabilized and stained with exogenously added iFluor phalloidin647. A high degree of colocalization was observed for iFluor phalloidin488 and phalloidin647. Further verifying the efficient cytosolic release of iFluor phalloidin488, proving the feasibility of performing colabeling experiments after PPSV_SS_ mediated delivery, and showing that almost all cytosolic filaments can be accessed by nanocontainer‐delivered phalloidin (Figure 4B). Similar data were obtained for primary human endothelial cells (HUVEC) (Figure 4C). We next verified whether PPSV_ss_ can be employed for the intracellular delivery of hydrophilic membrane‐impermeant drugs that are known to exert a strong biological effect. Therefore, we chose *α*‐amanitin, a membrane‐impermeable cyclic oligopeptide widely used to inhibit cell proliferation by inhibiting RNA polymerase II.^[^ ^52^, ^53^, ^54^ ^]^ Following treatment with PPSV_SS, amanitin_ both, HeLa cells and HUVEC show a marked inhibition of cell proliferation which was dependent on PPSV_ss_ encapsulation as the free drug showed no effect at the same concentrations (Figure S14, Supporting Information). Together, these results suggest that the PPSV_SS_ are first internalized into the endosomal system before the reductive environment and enzymatic digestion of peptides in endosomes triggers disintegration of the dual‐sensitive polypeptide shell and cyclodextrin core. The degraded polypeptide shell remains trapped in endosomes whereas the hydrophilic cargo, that is, pyranine or fluorescently labeled phalloidin, which is released from the disintegrated PPSV_SS_, escapes from endosomes into the cell's cytosol.

## 3 Conclusion

We synthesized and characterized dual‐responsive short (SPSV_SS_) and long polypeptide‐shelled nanocontainers (PPSV_SS_) based on amphiphilic cyclodextrins and demonstrated that the length of the peptide shell is crucial for successful and stable encapsulation of cargo. The PPSV_SS_ were able to deliver different types of hydrophilic and cell‐impermeable cargo (pyranine, phalloidin, *α*‐amanitin) to the intracellular milieu. PPSV_SS_ were efficiently internalized into endosomes of primary HUVEC and HeLa where the incorporated hydrophilic cargo was released via reductive and peptidase‐triggered disintegration of the PPSV_SS_. Thus, PPSV_SS_ serve as a novel biodegradable tool to deliver hydrophilic compounds into the intracellular cytosolic environment.

## 4 Experimental Section

Methods including DLS, cryo‐TEM, and light microscopic imaging as well as the synthesis of SPSV precursors and self‐assembly of PPSV_SS_ are outlined in the Supporting Information.

## Conflict of Interest

The authors declare no conflict of interest.

## Supporting information

Supporting Information

## Data Availability

Data available on request from the authors.

## References

[1] S. D. Koker, R. Hoogenboom, B. G. D. Geest, Chem. Soc. Rev. 2012, 41, 2867. 22282265 10.1039/c2cs15296g

[2] V. P. Torchilin, Nat. Rev. Drug Discovery 2014, 13, 813. 25287120 10.1038/nrd4333 PMC4489143

[3] C. Hofmann, A. Duerkop, A. J. Baeumner, Angew. Chem., Int. Ed. 2019, 58, 12840. 10.1002/anie.201811821 30633433

[4] L. Schoonen, J. C. M. van Hest, Adv. Mater. 2016, 28, 1109. 26509964 10.1002/adma.201502389

[5] J. Li, Y. Anraku, K. Kataoka, Angew. Chem., Int. Ed. 2020, 59, 13526. 10.1002/anie.202004180 32383236

[6] A. M. Reichmuth, M. A. Oberli, A. Jaklenec, R. Langer, D. Blankschtein, Ther. Delivery 2016, 7, 319. 10.4155/tde-2016-0006 PMC5439223 27075952

[7] A. C. Rice‐Ficht, A. M. Arenas‐Gamboa, M. M. Kahl‐McDonagh, T. A. Ficht, Curr. Opin. Microbiol. 2010, 13, 106. 20079678 10.1016/j.mib.2009.12.001

[8] W. C. de Vries, S. Kudruk, D. Grill, M. Niehues, A. L. L. Matos, M. Wissing, A. Studer, V. Gerke, B. J. Ravoo, Adv. Sci. 2019, 6, 1901935. 10.1002/advs.201901935 PMC6918114 31871866

[9] L. Sercombe, T. Veerati, F. Moheimani, S. Y. Wu, A. K. Sood, S. Hua, Front. Pharmacol. 2015, 6, 286. 26648870 10.3389/fphar.2015.00286 PMC4664963

[10] T. M. Allen, P. R. Cullis, Adv. Drug Delivery Rev. 2013, 65, 36. 10.1016/j.addr.2012.09.037 23036225

[11] N. Lamichhane, T. Udayakumar, W. D'Souza, C. Simone II, S. Raghavan, J. Polf, J. Mahmood, Molecules 2018, 23, 288. 10.3390/molecules23020288 PMC6017282 29385755

[12] E. Beltrán‐Gracia, A. López‐Camacho, I. Higuera‐Ciapara, J. B. Velázquez‐Fernández, A. A. Vallejo‐Cardona, Cancer Nanotechnol. 2019, 10, 11.

[13] S. Pottanam Chali, B. J. Ravoo, Angew. Chem., Int. Ed. 2020, 59, 2962. 10.1002/anie.201907484 PMC7028112 31364243

[14] J. Wang, D. Zhang, Adv. Polym. Technol. 2013, 32, E323.

[15] J. Hotz, W. Meier, Adv. Mater. 1998, 10, 1387.

[16] A. Samanta, M. Tesch, U. Keller, J. Klingauf, A. Studer, B. J. Ravoo, J. Am. Chem. Soc. 2015, 137, 1967. 25599114 10.1021/ja511963g

[17] W. C. de Vries, D. Grill, M. Tesch, A. Ricker, H. Nüsse, J. Klingauf, A. Studer, V. Gerke, B. J. Ravoo, Angew. Chem., Int. Ed. 2017, 56, 9603. 10.1002/anie.201702620 28485535

[18] N. U. Deshpande, M. Jayakannan, Biomacromolecules 2018, 19, 3572. 29906389 10.1021/acs.biomac.8b00833

[19] P. F. Monteiro, A. Travanut, C. Conte, C. Alexander, Wiley Interdiscip. Rev.: Nanomed. Nanobiotechnol. 2021, 13, e1678. 33155421 10.1002/wnan.1678

[20] J. Liu, B. Chang, Q. Li, L. Xu, X. Liu, G. Wang, Z. Wang, L. Wang, Adv. Sci. 2019, 6, 1801987. 10.1002/advs.201801987 PMC6446919 31139556

[21] M.‐F. Tsai, Y.‐L. Lo, Y. Soorni, C.‐H. Su, S. S. Sivasoorian, J.‐Y. Yang, L.‐F. Wang, ACS Appl. Bio Mater. 2021, 4, 3264. 10.1021/acsabm.0c01621 35014413

[22] J. Peng, Y. Liu, M. Zhang, F. Liu, L. Ma, C.‐Y. Yu, H. Wei, J. Controlled Release 2021, 334, 290. 10.1016/j.jconrel.2021.04.027 33905803

[23] M. Chen, D. Liu, F. Liu, Y. Wu, X. Peng, F. Song, J. Controlled Release 2021, 332, 269. 10.1016/j.jconrel.2021.02.030 33662455

[24] H. J. Forman, H. Zhang, A. Rinna, Mol. Aspects Med. 2009, 30, 1. 18796312 10.1016/j.mam.2008.08.006 PMC2696075

[25] B. Arunachalam, U. T. Phan, H. J. Geuze, P. Cresswell, Proc. Natl. Acad. Sci. U. S. A. 2000, 97, 745. 10639150 10.1073/pnas.97.2.745 PMC15401

[26] B. J. Ravoo, R. Darcy, Angew. Chem., Int. Ed. 2000, 39, 4324. 10.1002/1521-3773(20001201)39:23<4324::AID-ANIE4324>3.0.CO;2-O 29711925

[27] G. L. Ellman, Arch. Biochem. Biophys. 1958, 74, 443. 13534673 10.1016/0003-9861(58)90014-6

[28] C. K. Riener, G. Kada, H. J. Gruber, Anal. Bioanal. Chem. 2002, 373, 266. 12110978 10.1007/s00216-002-1347-2

[29] S. P. Chali, B. J. Ravoo, Macromol. Rapid Commun. 2020, 41, 2000049. 10.1002/marc.202000049 32419159

[30] J. Voskuhl, T. Fenske, M. C. A. Stuart, B. Wibbeling, C. Schmuck, B. J. Ravoo, Chem. ‐ Eur. J. 2010, 16, 8300. 20593445 10.1002/chem.201000623

[31] N. Düzgünes, S. Nir, Adv. Drug Delivery Rev. 1999, 40, 3. 10.1016/s0169-409x(99)00037-x 10837777

[32] G. Sahay, J. O. Kim, A. V. Kabanov, T. K. Bronich, Biomaterials 2010, 31, 923. 19853293 10.1016/j.biomaterials.2009.09.101 PMC3082844

[33] G. Sahay, D. Y. Alakhova, A. V. Kabanov, J. Controlled Release 2010, 145, 182. 10.1016/j.jconrel.2010.01.036 PMC2902597 20226220

[34] O. Harush‐Frenkel, N. Debotton, S. Benita, Y. Altschuler, Biochem. Biophys. Res. Commun. 2007, 353, 26. 17184736 10.1016/j.bbrc.2006.11.135

[35] H. Meng, S. Yang, Z. Li, T. Xia, J. Chen, Z. Ji, H. Zhang, X. Wang, S. Lin, C. Huang, Z. H. Zhou, J. I. Zink, A. E. Nel, ACS Nano 2011, 5, 4434. 21563770 10.1021/nn103344k PMC3125420

[36] O. Lunov, T. Syrovets, C. Loos, J. Beil, M. Delacher, K. Tron, G. U. Nienhaus, A. Musyanovych, V. Mailänder, K. Landfester, T. Simmet, ACS Nano 2011, 5, 1657. 21344890 10.1021/nn2000756

[37] K. T. Hastings, P. Cresswell, Antioxid. Redox Signaling 2011, 15, 657. 10.1089/ars.2010.3684 PMC3125571 21506690

[38] J. Yang, H. Chen, I. R. Vlahov, J.‐X. Cheng, P. S. Low, Proc. Natl. Acad. Sci. USA 2006, 103, 13872. 16950881 10.1073/pnas.0601455103 PMC1564263

[39] M. Felberbaum‐Corti, E. Morel, V. Cavalli, F. Vilbois, J. Gruenberg, PLoS One 2007, 2, e1144. 17987124 10.1371/journal.pone.0001144 PMC2043495

[40] K. Palande, O. Roovers, J. Gits, C. Verwijmeren, Y. Iuchi, J. Fujii, B. G. Neel, R. Karisch, J. Tavernier, I. P. Touw, J. Cell Sci. 2011, 124, 3695. 22045733 10.1242/jcs.089656 PMC3215578

[41] E. Friedmann, E. Hauben, K. Maylandt, S. Schleeger, S. Vreugde, S. F. Lichtenthaler, P.‐H. Kuhn, D. Stauffer, G. Rovelli, B. Martoglio, Nat. Cell Biol. 2006, 8, 843. 16829952 10.1038/ncb1440

[42] A. Pišlar, A. Mitrović, J. Sabotič, U. P. Fonović, M. P. Nanut, T. Jakoš, E. Senjor, J. Kos, PLoS Pathog. 2020, 16, e1009013. 33137165 10.1371/journal.ppat.1009013 PMC7605623

[43] B. M. Chain, P. Free, P. Medd, C. Swetman, A. B. Tabor, N. Terrazzini, J. Immunol. 2005, 174, 1791. 15699105 10.4049/jimmunol.174.4.1791

[44] M.‐A. Yessine, J.‐C. Leroux, Adv. Drug Delivery Rev. 2004, 56, 999. 10.1016/j.addr.2003.10.039 15066757

[45] B. J. Hong, A. J. Chipre, S. T. Nguyen, J. Am. Chem. Soc. 2013, 135, 17655. 24000948 10.1021/ja404491r PMC3916954

[46] K. Ray, B. Marteyn, P. J. Sansonetti, C. M. Tang, Nat. Rev. Microbiol. 2009, 7, 333. 19369949 10.1038/nrmicro2112

[47] E. J. Kwon, S. Liong, S. H. Pun, Mol. Pharmaceutics 2010, 7, 1260. 10.1021/mp1000668 PMC3018701 20476763

[48] J. S. Wadia, R. V. Stan, S. F. Dowdy, Nat. Med. 2004, 10, 310. 14770178 10.1038/nm996

[49] E. J. Kwon, J. M. Bergen, I. K. Park, S. H. Pun, J. Controlled Release 2008, 132, 230. 10.1016/j.jconrel.2008.06.012 PMC2695844 18627784

[50] M. An, D. Wijesinghe, O. A. Andreev, Y. K. Reshetnyak, D. M. Engelman, Proc. Natl. Acad. Sci. USA 2010, 107, 20246. 21048084 10.1073/pnas.1014403107 PMC2996653

[51] S. R. Sarker, M. Takikawa, S. Takeoka, ACS Appl. Bio Mater. 2020, 3, 2048. 10.1021/acsabm.9b01167 35025326

[52] K. V. der Jeught, H.‐C. Xu, Y.‐J. Li, X.‐B. Lu, G. Ji, World J. Gastroenterol. 2018, 24, 3834. 30228778 10.3748/wjg.v24.i34.3834 PMC6141340

[53] C. D. Kaplan, K.‐M. Larsson, R. D. Kornberg, Mol. Cell 2008, 30, 547. 18538653 10.1016/j.molcel.2008.04.023 PMC2475549

[54] V. T. Nguyen, F. Giannoni, M.‐F. Dubois, S.‐J. Seo, M. Vigneron, C. Kédinger, O. Bensaude, Nucleic Acids Res. 1996, 24, 2924. 8760875 10.1093/nar/24.15.2924 PMC146057

