# Supplementary material for: Biodegradable and Dual‐Responsive Polypeptide‐Shelled Cyclodextrin‐Containers for Intracellular Delivery of Membrane‐Impermeable Cargo
Source: Adv Sci (Weinh). 2021 Jul 18;8(18):2100694. doi: 10.1002/advs.202100694 (PMC8456233; doi:10.1002/advs.202100694)
Supplement: Supplementary file 1 — Supporting Information [file ADVS-8-2100694-s001.pdf]

© 2021 Wiley-VCH GmbH

ADVANCED SCIENCE
Open Access

Supporting Information

for *Adv. Sci.*, DOI: 10.1002/advs.202100694

Biodegradable and Dual-Responsive Polypeptide-Shelled Cyclodextrin-Containers for Intracellular Delivery of Membrane-Impermeable Cargo

Sergej Kudruk[†+], Sharafudheen Pottanam Chali[‡+], Anna Livia Linard Matos[†], Cole Bourque[§], Clara Dunker[†], Christos Gatsogiannis[§], Bart Jan Ravoo[‡] and Volker Gerke[†*]*

Supporting Information

Biodegradable and Dual-Responsive Polypeptide-Shelled Cyclodextrin-Containers for Intracellular Delivery of Membrane-Impermeable Cargo

Sergej Kudruk [†+], *Sharafudheen Pottanam Chali* [‡+], *Anna Livia Linard Matos* [†], *Cole Bourque* [§], *Clara Dunker* [†], *Christos Gatsogiannis* [§], *Bart Jan Ravoo* [‡*] *and Volker Gerke* [†*]

[†] S. Kudruk, A. L. L. Matos, C. Dunker, Prof. Dr. V. Gerke
Institute of Medical Biochemistry, Center for Molecular Biology of Inflammation, Westfälische Wilhelms Universität Münster, Von-Esmarch-Str. 56, 48149, Germany
E-mail: gerke@uni-muenster.de

[‡] Sharafudheen Pottanam Chali, Prof. Dr. B.J. Ravoo
Center for Soft Nanoscience and Organic Chemistry Institute, Westfälische Wilhelms Universität Münster, Busso Peus Straße 10, 48149, Germany
E-mail: b.j.ravoo@uni-muenster.de

[§] Cole Bourque, Prof. Dr. Christos Gatsogiannis
Center for Soft Nanoscience and Institute of Medical Physics and Biophysics, Westfälische Wilhelms Universität Münster, Busso Peus Straße 10, 48149, Germany and Max Planck Institute of Molecular Physiology, Otto Hahn Str. 11, 44227, Dortmund, Germany

[+] Equal contribution

[*] Corresponding authors

Instrumentation

^{1}H-NMR and ^{13}C-NMR spectra were recorded on an *DPX 300* (*Bruker*), Avance II 300 (*Bruker*), *Avance II 400 (Bruker)* or a DD2 600 instrument (*Agilent*). Chemical shifts δ in ppm are referenced to the solvent residual peak. **HRMS (ESI)** was performed using a MicroTOF ESI (*Bruker*) and an Orbitrap LTQ XL (*Thermo Scientific*). **Dynamic light scattering (DLS) and ζ-potential** measurements were carried out on a Nano ZS Zetasizer (*Malvern Instruments*) at 25 °C and samples were prepared in disposable 1 mL semi-micro PMMA cuvettes (*BRAND*) or in disposable DTS 1070 capillary cells (*Malvern Instruments*). Data analysis was performed with Zetasizer Software Version 7.12 (*Malvern Instruments*) and OriginPro 9.6.0172 (*Origin*). **Negative Stain Transmission electron microscopy.** 4µl sample droplets of CDV and PPSV's (in TBS buffer) were applied to freshly glow-discharged copper grids (Agar scientific; G2400C) covered by a continuous carbon film. For the CDV sample, the grids were first pre-treated with 0.1% w/v poly-lysine solution. The sample was left for 90 sec on the grid before blotting, using filter paper (Whatman no. 4). After staining with 0.75% uranyl formate for 75 sec, the grid was air-dried and transferred to a JEOL JEM-1400 electron microscope equipped with a LAB_6 cathode, operating at an acceleration voltage of 120kV. Digital micrographs were recorded at low dose conditions using a 4k × 4 k CMOS camera F416 (TVIPS, GmbH) at a magnification of 40,000 (pixel size 2.81 Å). Large view TEM images are reported in Figure S10. **Cryo-Electron Microscopy (cryoEM).** For cryoEM, 4 µl of each sample at an overall concentration of 20 mg/ml were applied to freshly glow discharged holey carbon Quantifoil 2/1 grids. The grid was then blotted after one minute incubation time and plunged into liquid ethane using a FEI Vitrobot Mark II system, 100% humidity, blotting time of 5.0 seconds at 4°C. Images were collected on a Talos Arctica cryoTEM (ThermoFisher) equipped with Volta phase plate (VPP) and Falcon III direct detector. Images were recorded in the automated acquisition program EPU (Thermo Fisher) in low dose conditions at a magnification of 120,000x corresponding to a pixel size of 1.2Å. Representative cryoEM images are shown in Figure S9.

Materials

All chemicals were purchased from *Sigma Aldrich*, *Merck*, *VWR*, *Carbolution*, *ABCR*, *Acros Organics*, *Alfa Aesar*, *Iris Biotech* or *TCI* and used as delivered unless otherwise stated. Dulbeccos phosphate buffered saline (DPBS, 1.47 mM KH_2PO_4, 7.67 mM Na_2HPO_4, 136.9 mM NaCl, 2.7 mM KCl (all *Sigma Aldrich*), pH 7.4) was prepared using ultrapure water with a resistance higher than 18 MΩ. HEPES buffer was prepared using 20 mM 4-(2-Hydroxyethyl)piperazine-1-ethanesulfonic acid (HEPES), 150 mM NaCl and ultrapure water, pH was adjusted to 7.4. For cell experiments sterile filtered DPBS with the same composition was purchased from *Sigma Aldrich*.

General Procedures

Preparation of cyclodextrin vesicles (CDV): Unilamellar bilayer vesicles of amphiphilic cyclodextrin derivatives were prepared by hydration of a thin film and subsequent extrusion. The solvent was evaporated in a stream of argon to obtain a thin film and residual solvent was removed under high vacuum. The film was hydrated by addition of HEPES to yield a total amphiphile concentration of 100 µM. For loaded containers CDV_{cargo}, pyranine (5 mM), phalloidin488 (20 µM) or α-amanitin (0.3 mM) solution in HEPES was added and the amphiphilic cyclodextrin film was hydrated. After vigorous stirring overnight, this solution was vortexed, and repeatedly passed through a polycarbonate membrane with 100 nm pore size (*AVESTIN*) in a Liposofast manual extruder (*AVESTIN*) to yield CDV.

Preparation of Short-peptide shelled vesicles ($SPSV_{SS}$): For the case of Ad-GGCCDD, Ad-TEG-GGCCDD or Ad-GGGCCCDDD, 25 µL of 2 mM Ad-GGCCDD or Ad-TEG-GGCCDD or Ad-GGGCCCDDD in HEPES buffer was added to 1 mL 100 µM CDV and stirred for 15 min to give $SPSV_{SH}$. After stirring in an open vial for aerial oxidation overnight $SPSV_{SS}$ were obtained. For the case of Ad-GGGDDDD, 25 µL of 2 mM Ad-GGGDDDD in HEPES buffer was added to 1 mL CDV and stirred for 15 min to give $SPSV_{COOH}$. 1.6 µL of 1 M EDC.HCl in HEPES buffer was added to $SPSV_{COOH}$ and stirred for 30 min followed by 2 µL of 50 mM of cystamine in HEPES buffer and stirred overnight to obtain $SPSV_{SS}$.

Preparation of polypeptide shelled vesicles ($PPSV_{SS}$): 25 µL of 2 mM Ad-PLG_{105} in HEPES buffer was added to 1 mL CDV and stirred for 30 min to yield $PPSV_{COOH}$. 50 µL of 1 M EDC·HCl in HEPES buffer was added to $PPSV_{COOH}$ and stirred for 30 min followed by 25 µL of 50 mM of cystamine in HEPES buffer and stirred overnight. Non-encapsulated dyes were separated by gel filtration.

Preparation of Dy633 conjugated $PPSV_{SS}$: 25 µL of 2mM Ad-PLG_{105} in HEPES buffer was added to 1 mL 100 µM CDV and stirred for 30 min to yield $PPSV_{COOH}$. 50 µL of 1 M EDC·HCl in HEPES buffer was added to $PPSV_{COOH}$ and stirred for 30 min followed by addition of 25 µL of 50 mM of cystamine in HEPES buffer and stirred overnight. 20 µL of 5 mM Dy633 was added to this solution and stirred for 6 h. Non-conjugated dye and other reagents were removed by dialysis against HEPES buffer.

Cell experiments

Cell culture: Primary human umbilical vein endothelial cells (HUVEC), purchased from *PromoCell* (C-12203), were cultured at 37 °C and 5 % CO_2 for up to 5 passages on CellBIND plates (*Corning*, CLS3296-40EA) in HUVEC mix medium consisting of Endothelial Cell Growth Medium 2 (ECGM2, *PromoCell*, C-22011) and M199 medium containing 10% fetal calf serum (FCS) (*Sigma*, F7524) at a 1:1 ratio. The medium was further supplemented with 30 µg/mL gentamycin (*Sigma*, G1397) and 15 ng/mL amphotericin B (*Biochrom*, A2612). For microscopy, HUVEC were cultured in µ-slide 8 well glass bottom dishes (*ibidi*, 80287) pre-coated with collagen (type I). HeLa cells were cultured at 37 °C and 7 % CO_2 for up to 15 passages in high glucose DMEM (4500 mg/L glucose, Sigma-Aldrich) supplemented with 10% (v/v) fetal calf serum (FCS, Biochrom), 1% L-glutamine (Lonza), 100 U/mL penicillin (Lonza) and 100 µg/mL streptomycin (Lonza). HeLa cells were cultured in µ-slide 8 well glass bottom dishes (*ibidi*, 80287) for confocal microscopy in described medium.

Uptake: For analyzing the time dependent uptake, freshly prepared $PPSV_{SS}$ were dialyzed against M199, diluted 1:4 in serum free high glucose DMEM and added to HUVEC for the times indicated in a live cell experimental setup (37 °C and 5% CO_2). For all other uptake experiments, the freshly dialyzed $PPSV_{SS}$ were diluted in M199 and incubated with a HUVEC cell layer for 30 min at 37°C and 5% CO_2. After washing 5 times with M199, cell growth was continued in a 1:1 mixture of ECGM2 and M199 with 10% FCS. For uptake of $PPSV_{SS}$ in HeLa cells same procedure was used as described but the incubation setup was changed to 37 °C and 7% CO_2.

Costaining with endosome marker: For colocalization experiments, FITC dextran 10 kDa (Thermo Fisher Scientific) (dissolved in DMSO to 1 mM stock solution) was used. The marker was diluted to a 1 µM concentration and applied to the cells together with the $PPSV_{SS}$ for 30 min.

Immunoflurescence/Phalloidin staining: 2 h post uptake of the iFluor phalloidin488 loaded PPSVss, cells were washed four times with DPBS (Sigma Aldrich) and fixed with 4% (w/w) paraformaldehyde (PFA, Sigma Aldrich) in DPBS for 10 min, permeabilized in 0.5% (v/v) Triton X-100 (AppliChem) in DPBS for 10 min and washed three times with DPBS-T (DPBS, 0.1% (v/v) Triton X-100 (AppliChem)). After blocking for 30 minutes with 0.5% (w/w) bovine serum albumin (BSA, Roth) in DPBS, samples were counterstained either with iFluor phalloidin647 (Sigma Aldrich) or with EEA1 primary mouse antibodies for 1 h followed by incubation of a secondary anti-mouse antibody Alexa Fluor 594 for additonal 1 h at room temperature. For time dependent uptake DAPI (1 µg/mL, ThermoFischer Scientific) in DPBS was co-incubated at room temperature. Cells were washed with DPBS and fixed with Mowiol (Sigma-Aldrich) on glass slides. Confocal imaging was performed using the LSM 780 microscope (Carl Zeiss).

Live cell microscopy: Live-cell imaging employed a LSM 780 confocal laser scanning microscope (CLSM, *Carl Zeiss*) equipped with the objective lense Plan-Apochromat x 63/1.4 oil

and differential interference contrast objective lenses (*Carl Zeiss*) in a 37°C environment with 5 % CO_2. Data were processed using Fiji (*Nat. Methods*, **2012**, *9*, 676).

Lactate dehydrogenase (LDH) assay: Cytotoxicity of $PPSV_{SS}$ on HeLa cells or HUVEC was assessed by measuring the lactate dehydrogenase (LDH) activity released from damaged cells. The cells were seeded in 96-well plates with a density of 3000 cells/well and cultured for 1 d. Subsequently, the culture medium was replaced with assay medium containing different $PPSV_{SS}$ concentration, ranging from 0 to 50 µM in serum-free medium. The plates were left in the incubator either for 2 h or 4 h at 37°C. The maximal LDH activity that represents the control values was determined by addition of 1% Triton X-100 for 10 min to completely lyse the cells. After transfer of the cell supernatant to a new 96-well plate, they were mixed in a volume ratio of 1:1 with reaction mixtures containing the tetrazolium salt, 2-(4-iodophenyl)-3-(4-nitrophenyl)-5-phenyl-2H-tetrazolium (INT), followed by incubation for exact 30 min at 37°C, protected from light. The reaction was stopped by addition of 50 µL HCl (1 N) to each well. The values of relative toxicity (percentage of LDH activity as compared to the maximal value obtained after Triton treatment) were calculated after measuring the absorbance at 492 nm on a Microplate Reader. The concentration-response curve obtained was fitted to a growth-sigmoidal function. LDH assays were carried out for each sample in triplicate (n = 3).

Flow cytometric analysis of $PPSV_{SS,pyr}$ internalization: Cells were treated with $PPSV_{SS,pyr}$ for for the given amount of time at 37 °C. Cells were then washed in PBS (Sigma), and detached in accutase solution (Sigma) for 3 min at 37 °C. 7AAD (eBioscience, San Diego, CA, USA) allowed the exclusion of dead cells. A Guava easyCyte™ System (Millipore, Burlington, MA, USA) was used to determine the percentage of pyranine positive (excitation at 450 nm) cells per 10,000 cells. $PPSV_{SS}$ were used as a negative control. FACS assays were carried out for each sample in triplicate (n = 3).

Cell viability assay: The cytotoxicity of $PPSV_{SS,\ amanitin}$ was assessed in HeLa cells and HUVEC. Cells (2,000 cells/well) were added to the wells of a 96-well plate (Corning, Woburn, MA, USA). After culturing at 37 °C for 24 h, the cells were incubated with $PPSV_{SS,\ amantin}$ at either 0, 10, 20, 30, 40 or 50 µM (in M199 Medium) for 4h, washed and then cultivated for further 24 h. The proportion of viable cells was evaluated using a CCK-8 kit (Sigma-Aldrich, St. Louis, MO, catalog No. 96992) or phase contrast microscopy to count adherent viable cells after treatment of $PPSV_{SS,\ amanitin}$. Blank wells only with culture media and PBS-treated wells were used to define 0 and 100% viability, respectively. CCK-8 assays were carried out for each sample in triplicate (n = 3).

Chemical Synthesis

General: All reactions were carried out in heat-gun-dried glassware under argon atmosphere and were performed by using standard *Schlenk* techniques. Thin layer chromatography was carried out on *Merck* silica gel 60 F254 plates; detection by UV or dipping into a solution of $KMnO_4$ (1.5 g), $NaHCO_3$ (5.0 g) in H_2O (400 mL) followed by heating. Flash chromatography (FC) was carried out on *Merck* silica gel 60 (40 – 63 µm) at an argon pressure of 0-0.5 bar.

Synthesis of adamantane precursor for adamantane terminated short-peptides (Ad-TEG)

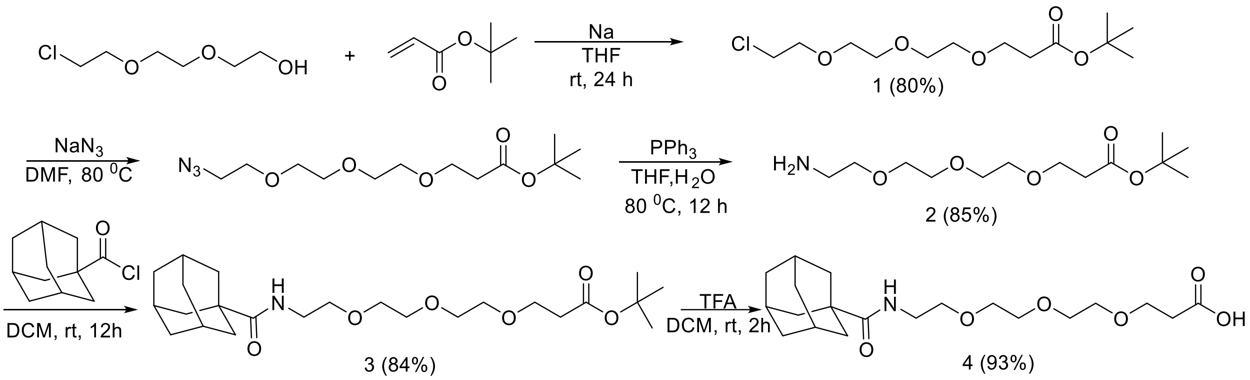

Scheme S1. Synthesis scheme for 1-((1r,3R,5S)-adamantan-1-yl)-1-oxo-5,8,11-trioxa-2-azatetradecan-14-oic acid. 2-[2-(2-Chloroethoxy)ethoxy]ethanol underwent Michael addition with tert-butyl acrylate in the presence of sodium to yield **1**. Chloride **1** is substituted with azide which is further reduced to amine to yield **2**. Reaction of amine on **2** with adamantane acid chloride yield **3**, which undergoes deprotection of acid in the presence of TFA to yield the desired compound **4.**

Short peptide synthesis

Synthesis of Ad-GGCCDD: Peptides were synthesized using solid phase peptide synthesis (SPPS). 2-chlorotrityl-resin (1.6 mmol/g) and Fmoc-Asp(OtBu)-OH (1.5 eq. relative to the amount of active functionalities on the resin) were dissolved in dry DCM (25 ml) under argon atmosphere. DIPEA (2 eq) was added and the mixture was agitated for 5 min by the argon stream. A second portion of DIPEA (3 eq) was added and then agitated for 2 h by the argon stream. Methanol (1 ml/g resin) was added and the resulting mixture was agitated for 15 min to quench the remaining resin functionalities. After filtration of the reaction mixture the resin was washed with DCM (3 x 30 ml), DMF (3 x 30 ml), DCM (3 x 30 ml) and methanol (3 x 30 ml). The resin was dried under vacuum. The elongation was performed using an automated peptide synthesizer. After transferring the dry resin to the reaction vessel, it was pre swollen by shaking in DMF (20 ml) for 5 min and then washed with DMF (2 x 20 ml) The Fmoc protecting group was cleaved by shaking in 20% piperidine solution in DMF (20 ml). After removing the solution another portion of 20% piperidine solution in DMF (20 ml) was added and shaken for 20 min to ensure complete deprotection. The resin was washed with DMF (7 x 20 ml) and the second Fmoc-protected amino-acid (Fmoc-ASP(OtBu)-OH) (3 eq. relative to resin loading, 0.5 M solution in DMF) was added. HOBt (4 eq, 0.4 M solution in DMF) and DIPCDI (4 eq, 0.4 M solution in DMF) were added and the mixture was shaken for 2.5 h. After washing with DMF (3 x 20 ml) the procedure was repeated for Fmoc-Cys(Trt)-OH two times, Fmoc-Gly-OH two times and with adamantane acid. After the completion of the addition of amino acids, the resin was suspended in a solution of TFA:H_2O:Triisopropylsilane:edt (95:2.5:2.5:1, 20 ml) and stirred overnight to cleave from the resin and to remove the protecting groups. The reaction mixture was sucked off and the resin was washed with TFA and peptides then precipitated by the addition of cold Et_2O. After collecting the precipitate by centrifugation, it was further purified by using RP-MPLC with the eluents 0.05% TFA in water and acetonitrile. Other peptides Ad-TEG-GGCCDD, Ad-GGGCCCDDD, Ad-GGGDDDD were synthesized accordingly. Product formation was confirmed by MALDI TOF or HRMS and the purity was also confirmed by RP-MPLC.

tert-butyl 3-(2-(2-(2-chloroethoxy)ethoxy)ethoxy)propanoate (1)

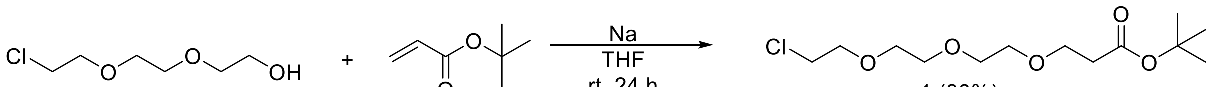

1 (80%)

2-(2-(2-chloroethoxy)ethoxy)ethan-1-ol (25 g, 148.26 mmol) in 50 ml dry THF was mixed with a catalytic amount of sodium under argon atmosphere and stirred until it dissolved completely. Tert-butyl acrylate (24.7 g, 192.74 mmol) was added and stirred for 24 h at room temperature. After completion of the reaction, monitored by TLC, 10 mL of 1 M HCl was added to the reaction mixture and stirred for 10 minutes. THF was removed under reduced pressure and the rest of the solution was suspended in 100 mL brine solution for 15 minutes. The product was extracted to ethyl acetate (3 x 80 mL) and pure product was obtained as a colourless liquid after column chromatography using ethyl acetate and cyclohexane as an eluent. (yield = 80%).

^{1}H NMR (400 MHz, $CDCl_3$, δ): 3.62 (m, 14H, C**H**$_2$O), 2.46 (t, 2H, C**H**$_2$CO), 1.4 (s, 9H, C**H**$_3$);

^{13}C NMR (100 MHz, $CDCl_3$, δ): 170.59, 80.18, 71.07, 70.38, 70.32, 70.29, 70.10, 66.62, 42.42, 35.99, 27.81

HRMS (ESI) *m/z*: $[M + Na]^+$ calcd for $C_{13}H_{25}ClO_5$, 319.1391; found, 319.1282.

tert-butyl 3-(2-(2-(2-aminoethoxy)ethoxy)ethoxy)propanoate (2)

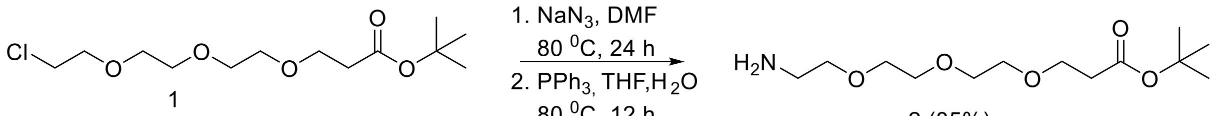

2 (85%)

A solution of **1** (5g, 16.84 mmol) in 20 mL dry DMF was heated at 80 °C and NaN_3 (3.29, 50.54 mmol) was added under argon atmosphere and heated at 80 °C for 24 h. After completion of the reaction the solvent was removed under reduced pressure, it was dissolved in 100 mL THF and the solid impurities were filtered out. PPh_3 (5.3 g, 20.21 mmol) was to the reaction mixture and stirred for 10 h. 10 mL water was added and then stirred for another 2 h. THF was evaporated completely, 50 mL water was added, and the precipitate was filtered. Water was removed under reduced pressure. The remaining byproducts were removed by precipitation in diethyl ether. The

product was obtained as a colorless liquid after the removal of solvent, which was used without any further purification. (yield = 85%)

^{1}H NMR (400 MHz, $CDCl_3$, δ): 3.66 (t, 2H, C**H**$_2$O), 3.58 (m, 8H, C**H**$_2$O), 3.47 (t, 2H, C**H**$_2$O), 2.83 (m, 2H, C**H**$_2$NH$_2$), 2.45 (t, 2H, C**H**$_2$CO), 1.78 (s, 2H, N**H**$_2$), 1.40 (m, 9H, C**H**$_3$).

^{13}C NMR (100 MHz, $CDCl_3$, δ): 170.97, 80.55, 73.21, 70.60, 66.93, 41.74, 36.29, 28.12.

HRMS (ESI) *m/z*: $[M + H]^+$ calcd for $C_{13}H_{27}NO_5$, 278.1889; found, 278.1972

tert-butyl 1-((1r,3R,5S)-adamantan-1-yl)-1-oxo-5,8,11-trioxa-2-azatetradecan-14-oate (**3**)

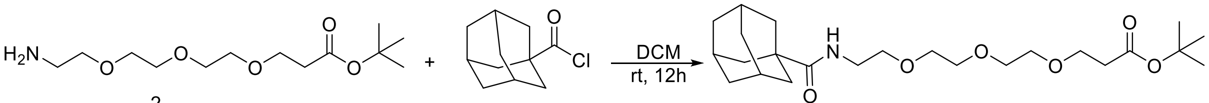

2 3 (84%)

1-Adamantanecarbonyl chloride (6 g, 21.63 mmol) in 10 mL dry DCM was added dropwise to a solution of **2** (3.85 g, 19.47 mmol) in 50 mL dry DCM at 0 °C and then stirred for 12h at room temperature. DCM was removed and the pure product was obtained as colourless viscous liquid after column chromatography using ethyl acetate and cyclohexane as eluents. (yield = 84%)

^{1}H NMR (400 MHz, $CDCl_3$, δ): 6.10 (s, 1H, CON**H**), 3.74(t, 2H, C**H**$_2$O), 3.61 (m, 8H, C**H**$_2$O), 3.52 (t, 2H, C**H**$_2$O), 3.43 (t, 2 H, C**H**$_2$O), 2.48 (t, 2H, C**H**$_2$CO), 2.01-1.60 (m, 15H, C**H**$_2$, C**H**), 1.42 (m, 9H, C**H**$_3$).

^{13}C NMR (100 MHz, $CDCl_3$, δ): 178.46, 171.28, 80.96, 70.93, 70.82, 70.67, 70.32, 67.35, 41.02, 39.61, 39.41, 36.97, 36.68, 28.52.

HRMS (ESI) *m/z*: $[M + Na]^+$ calcd for $C_{24}H_{41}NO_6$, 462.2934; found, 462.2846

1-((1r,3R,5S)-adamantan-1-yl)-1-oxo-5,8,11-trioxa-2-azatetradecan-14-oic acid (**4**)

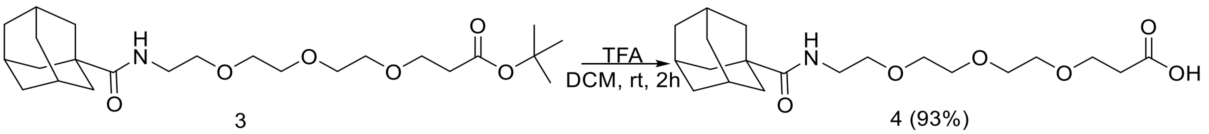

3 4 (93%)

Deprotection of the acid was carried out using TFA. In a typical procedure **3** (4 g) was dissolved in 20 mL DCM and 20 mL cold TFA was added slowly and then stirred for 2 h at room temperature. Solvent was completely evaporated and the product was purified by column chromatography using methanol and DCM as eluents. (yield = 93%)

^{1}H NMR (400 MHz, $CDCl_3$, δ): 10.73 (s, 1H, COO**H**), 6.33 (s, 1H, CON**H**), 3.74 (t, 2H, C**H**$_2$O), 3.62 (m, 8H, C**H**$_2$O), 3.55 (t, 2H, C**H**$_2$O), 3.44 (t, 2H, C**H**$_2$O), 2.60 (t, 2H, C**H**$_2$CO), 2.02-1.60 (m, 15H, C**H**$_2$, C**H**).

^{13}C NMR (100 MHz, $CDCl_3$, δ): 179.15, 175.34, 70.64, 70.48, 70.38, 70.26, 69.85, 66.58, 40.72, 39.39, 39.06, 36.53, 34.99, 28.14.

HRMS (ESI) *m/z*: $[M + Na]^+$ calcd for $C_{20}H_{33}NO_6$, 406.4850; found, 406.2210

NMR Spectra

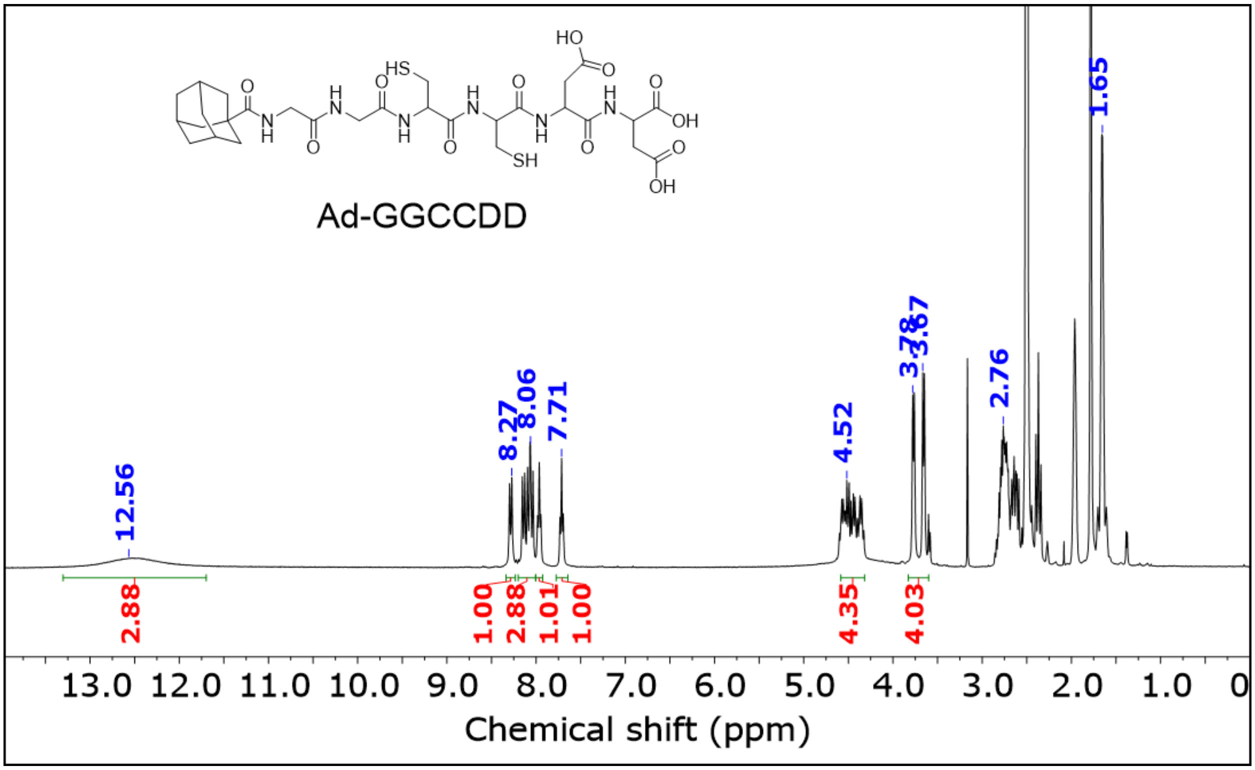

Figure **S1**. 1H-NMR of Ad-GGCCDD peptide (DMSO-d6).

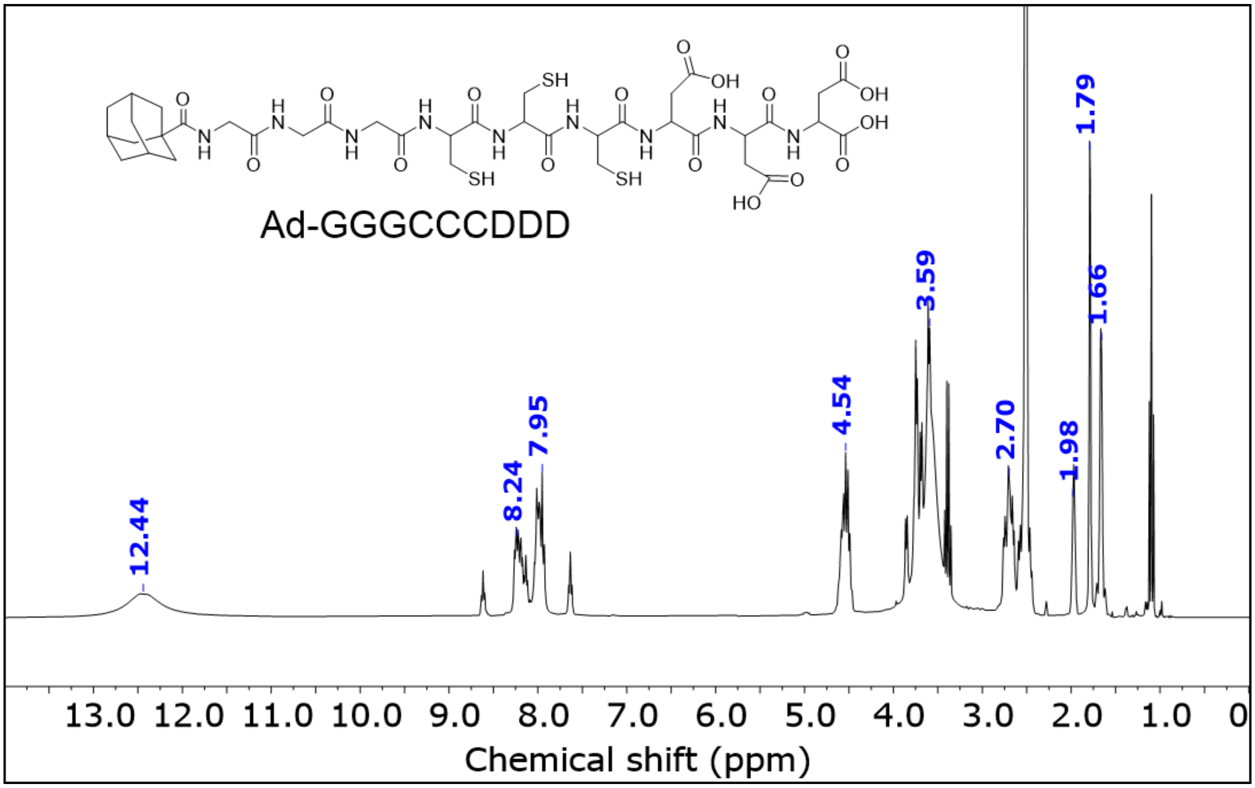

Figure **S2**. 1H-NMR of Ad-GGGCCCDDD peptide (DMSO-d_6).

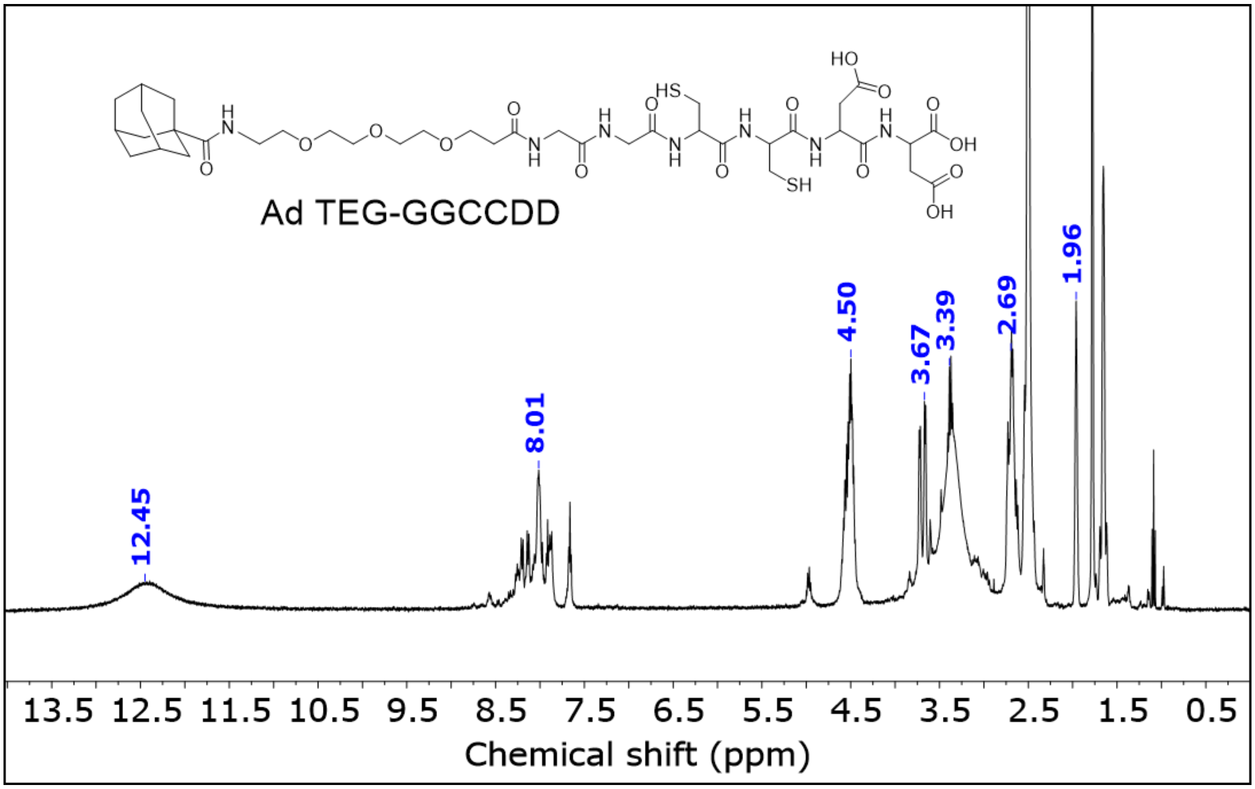

Figure **S3**. 1H-NMR of Ad-TEG-GGCCDD peptide (DMSO-d_6).

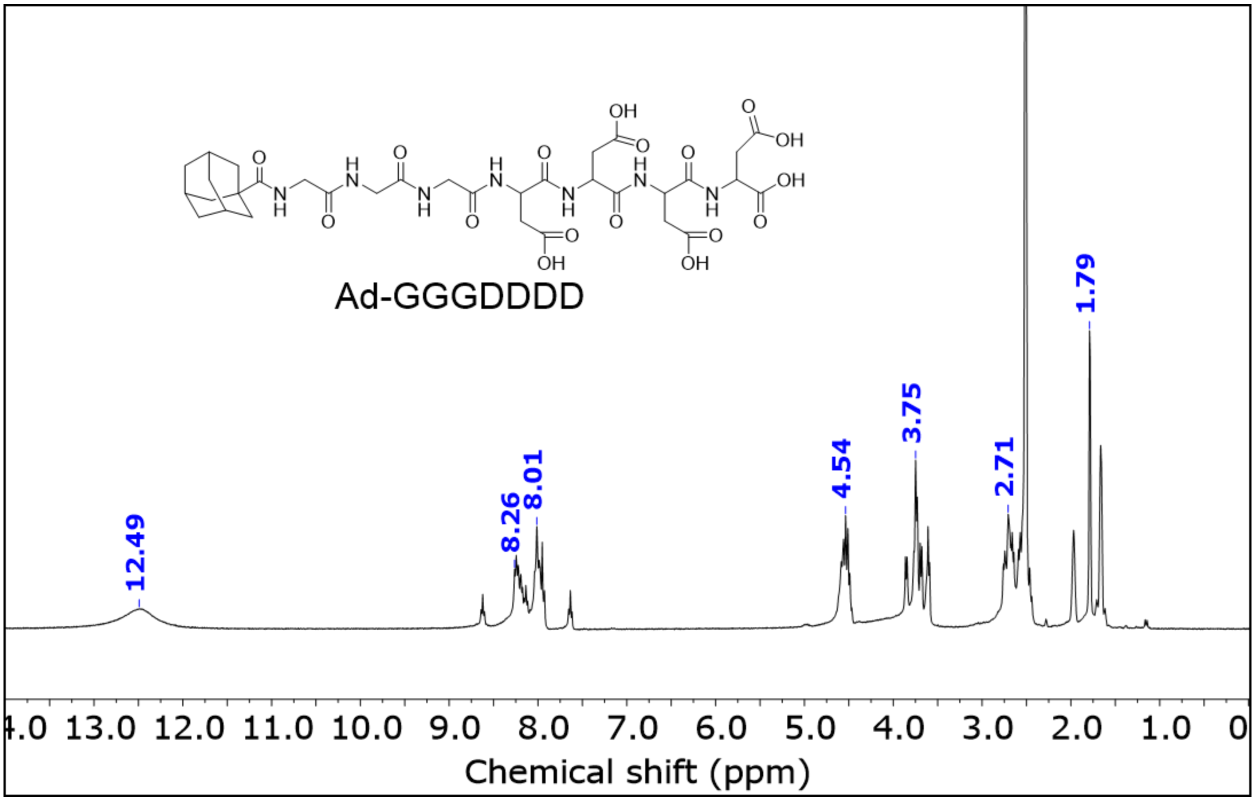

Figure **S4**. ^{1}H-NMR of Ad-GGGDDDD peptide (DMSO-d_6).

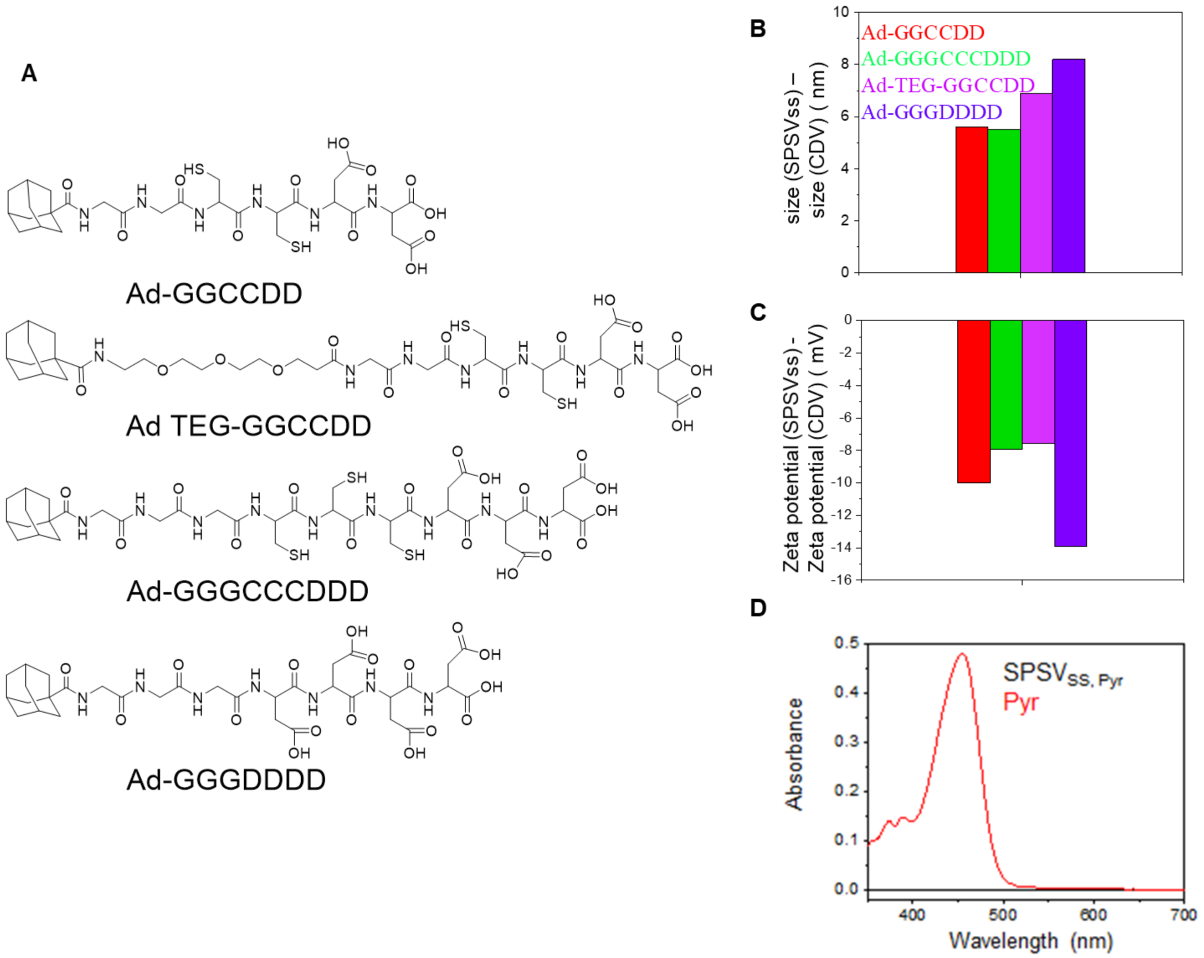

Figure S5. **A.** Peptide sequences used for the design of SPSV$_{SS}$. **B.** Size differences and **C.** zeta potential differences for SPSV$_{SS}$ formed from CDV using different short peptide shells. **D.** Pyranine and SPSV$_{SS,}$ pyr UV-Vis absorbance spectra of gel-filtrated SPSV$_{SS}$ after encapsulation of pyranine and oxidative crosslink of SPSV$_{SH}$, that contained the GGCCDD peptide sequence. Note the absence of the absorbance peak indicating that encapsulation was not sufficient enough.

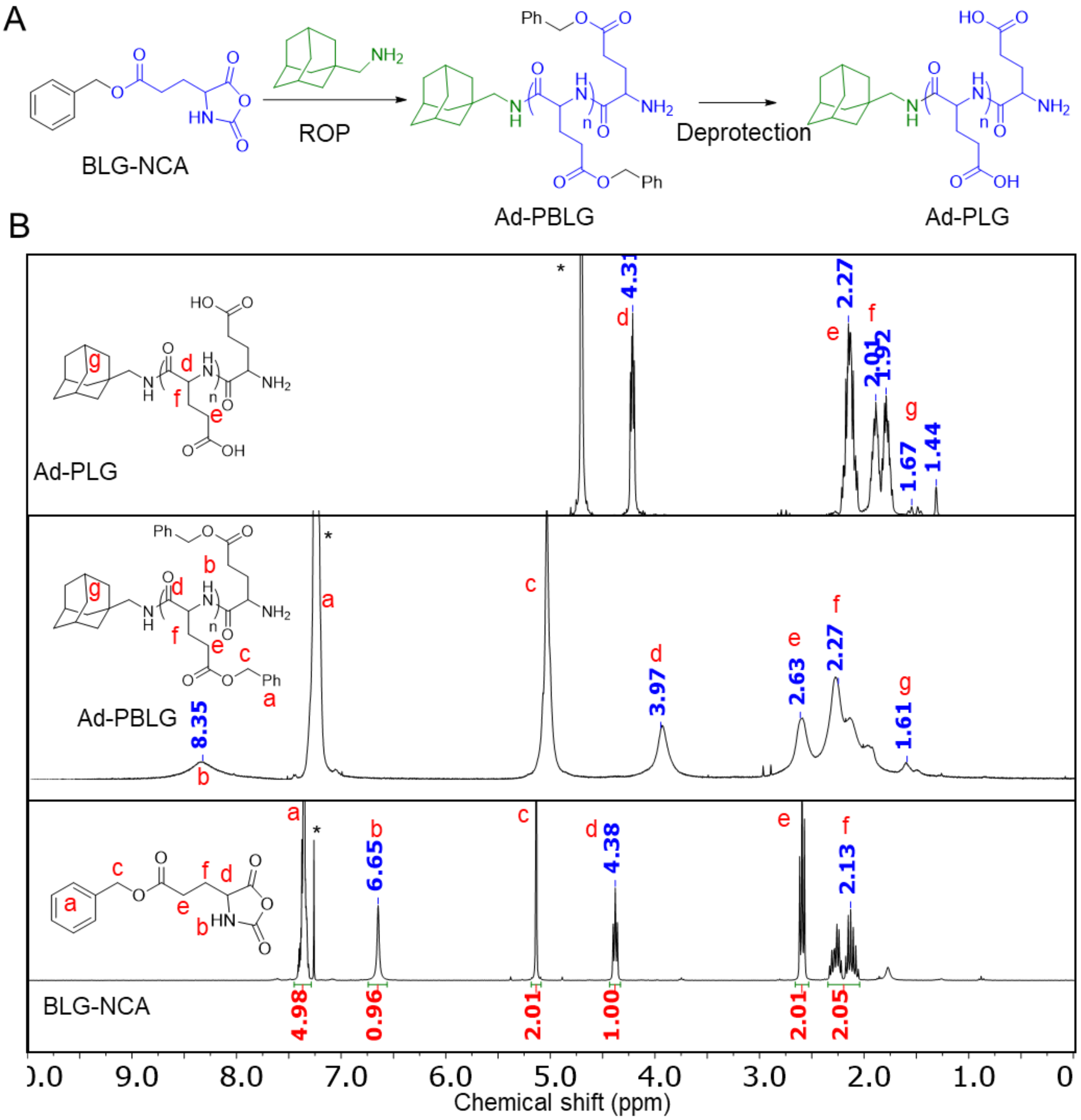

Figure **S6**. A. Scheme for the synthesis of Ad-PLG. ROP of the monomer BLG-NCA by Ad methyl amine gives Ad-PBLG, Ad-PLG is obtained after the benzyl protected acid groups on Ad-PBLG are deprotected. B. ^{1}H-NMR (400 MHz) comparison of BLG-NCA (in $CDCl_3$), Ad-PBLG (in $CDCl_3$) and Ad-PLG (in D_2O). [1]

Additional Experimental Data

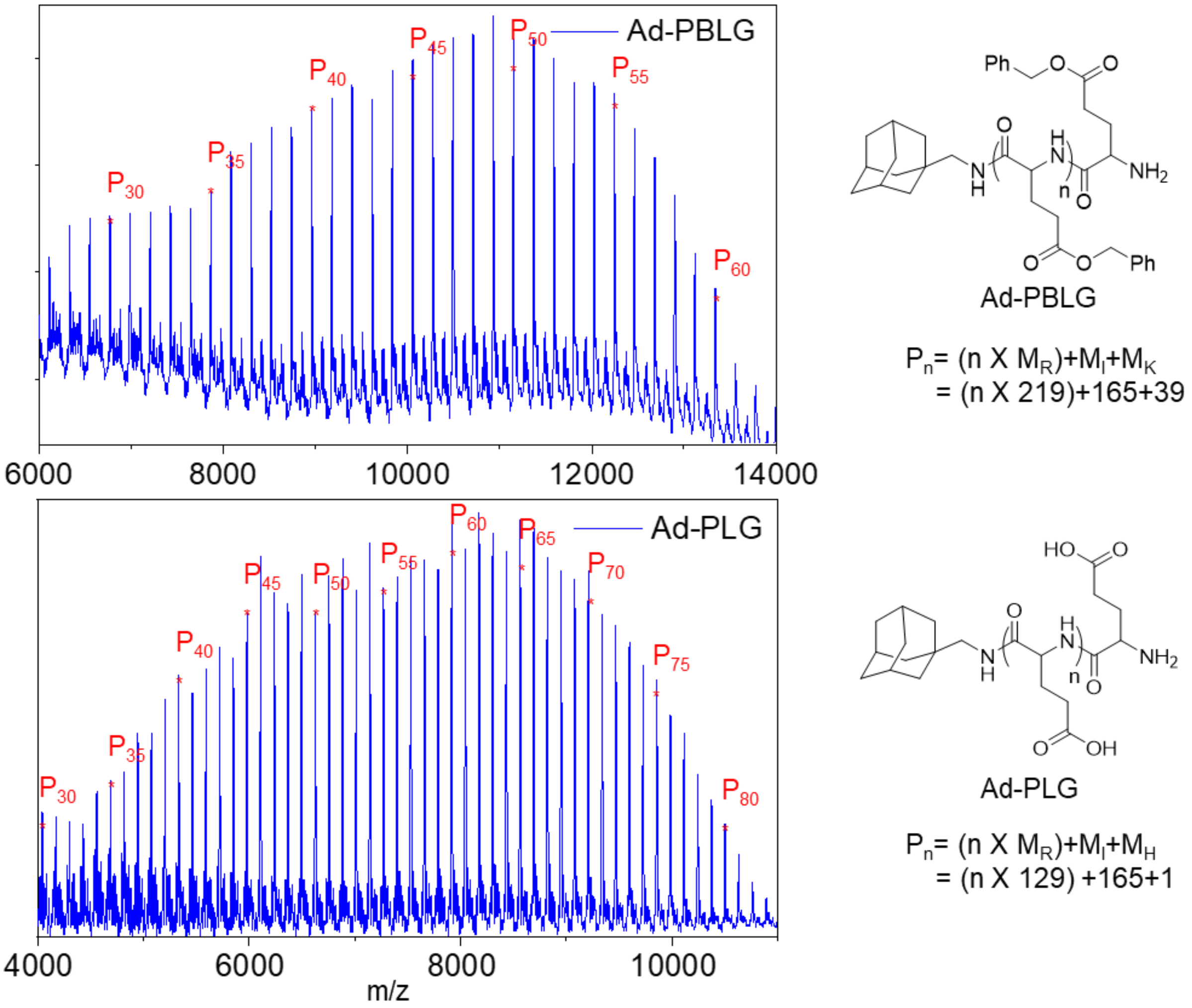

Figure **S7**. MALDI spectra of Ad-PBLG and Ad-PLG. M_R = repeating unit mass, n = number of repeating units, M_I = mass of initiator, M_k = mass of K^+, M_H = mass of H^+. [1]

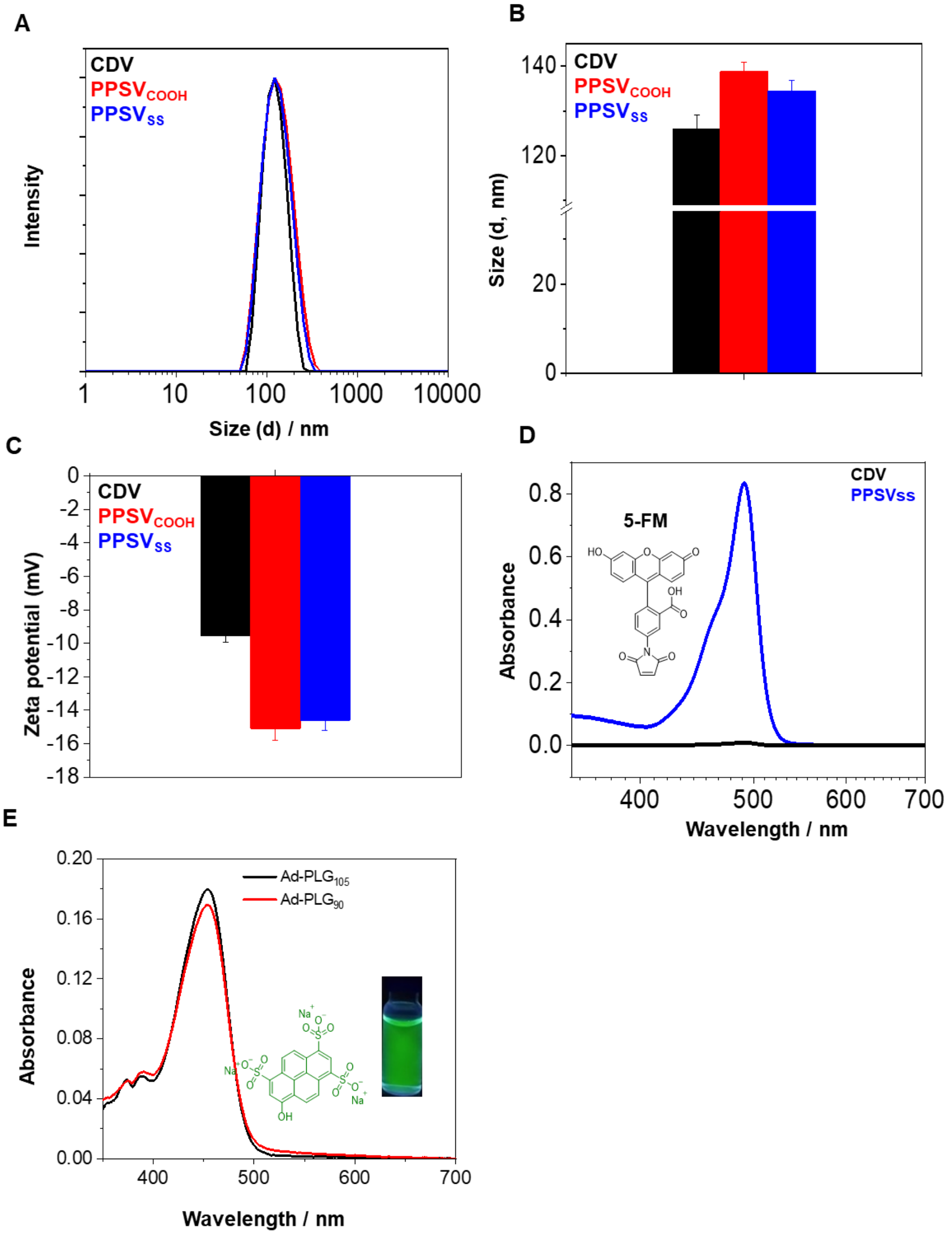

Figure S8. A. DLS analysis of the pyranine containing nanocontainers PPSVss. **B.** Bar diagram showing the size obtained for pyranine containing CDV, $PPSV_{COOH}$, and $PPSV_{SS}$ obtained by DLS. **C.** Bar diagram showing the zetapotential for pyranine containing CDV, $PPSV_{COOH}$, and $PPSV_{SS}$. **D.** Cystamine mediated crosslinking to form $PPSV_{SS}$. Quantification of crosslinking efficiency was performed using fluorescein-5-maleimide (5-FM) dye after reducing with tris(2-carboxyethyl)phosphine-hydrochloride (TCEP) where free thiols react with the 5-FM dye. The absorption spectrum was used to quantify the crosslinking efficiency (9 % for the $PPSV_{SS}$). **E.** Absorbance spectra of pyranine-containing PPSVss, $_{pyr}$ to quantify the amount of dye encapsulated.

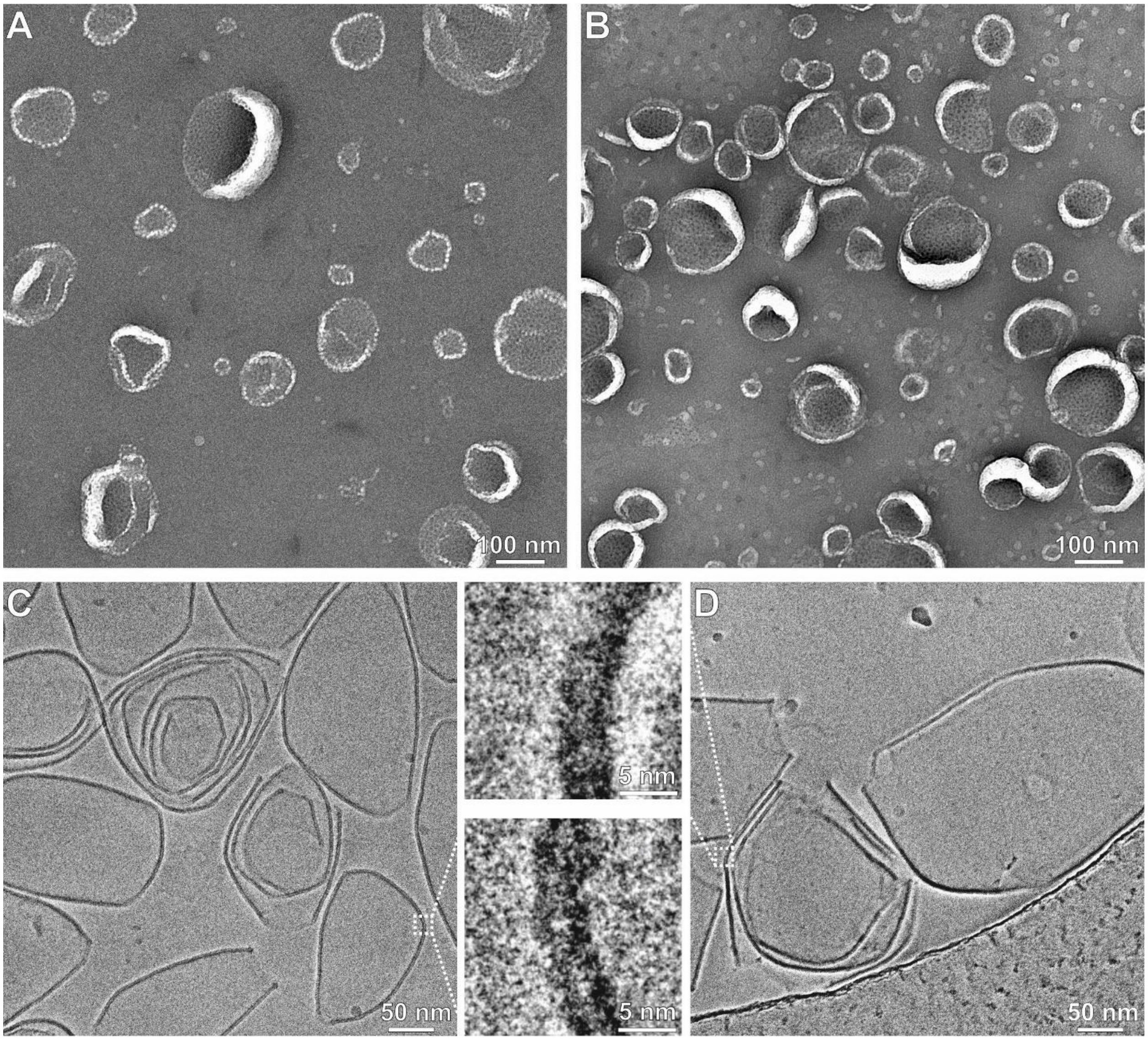

Figure S9. Negative stain TEM images of CDV (**A**) and PPSVss (**B**) (staining with uranyl formate). **C** and **D** show cryo-TEM images of CDV and PPSVss, respectively marked by the checked boxes are areas that have been zoomed to show the membranes.

Cell experiments

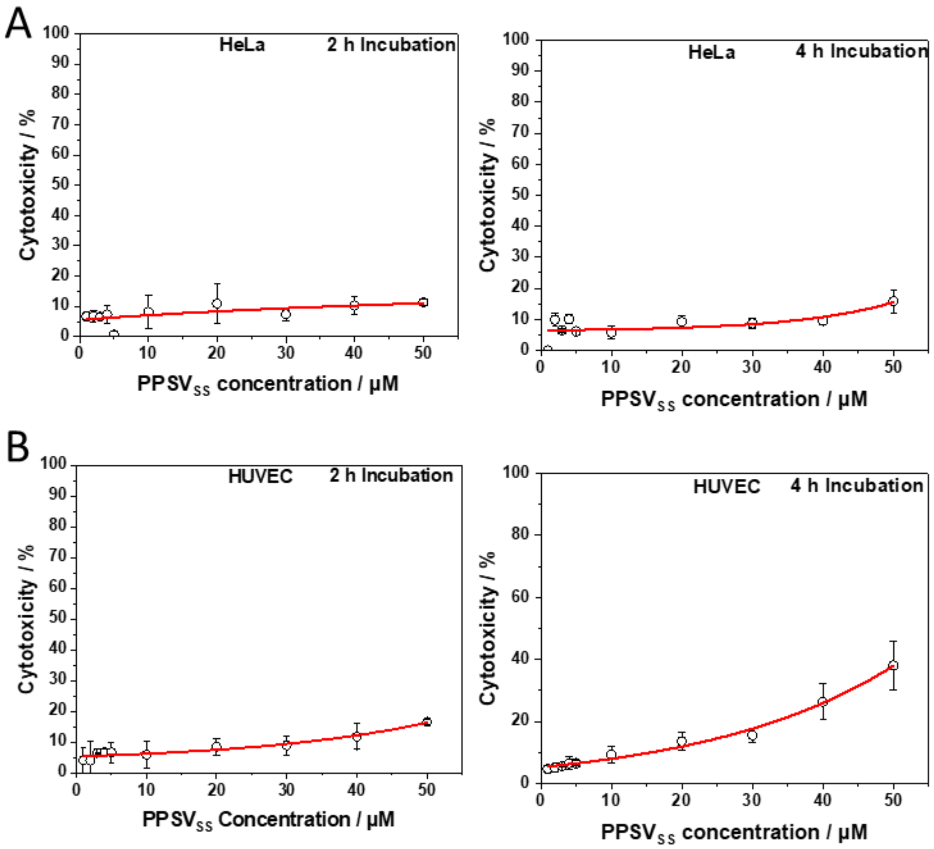

Figure S10. Cytotoxicity of PPSV$_{SS}$ on HeLa cells (**A**) and HUVEC (**B**) either after 2 h (left graphs) or 4h incubation (right graphs) was assessed by measuring the lactate dehydrogenase (LDH) activity. The assay was performed in serum-free medium with PPSV$_{SS}$ concentrations ranging between 0 and 50 µM. The cells were incubated at 37°C. The response curves were fitted to a growth sigmoidal function employing OriginPro 2021 (64-bit) 9.8.0.200. Mean cytotoxicity value ± SD are shown for individual concentrations obtained in three independent experiments.

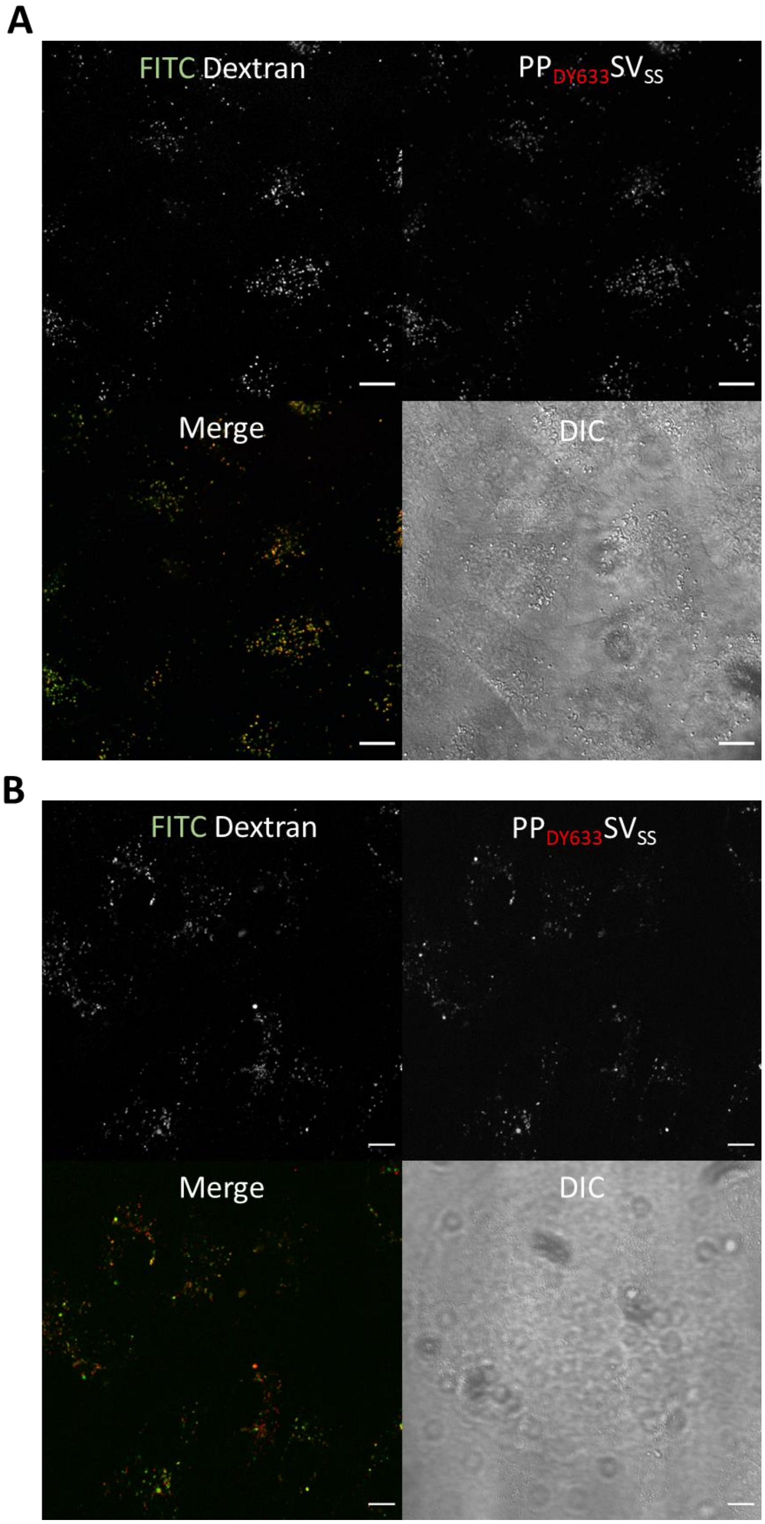

Figure S11. Intracellular uptake of the fluorescently shell-labeled $PP_{Dy633}SV_{SS}$ in HeLa cells (**A)** and HUVEC (**B)** analyzed via confocal microscope. Co-incubation was performed with fluorescein-conjugated dextran (FITC-Dextran, Mn ~ 10 kDa) appearing in punctuate structures.

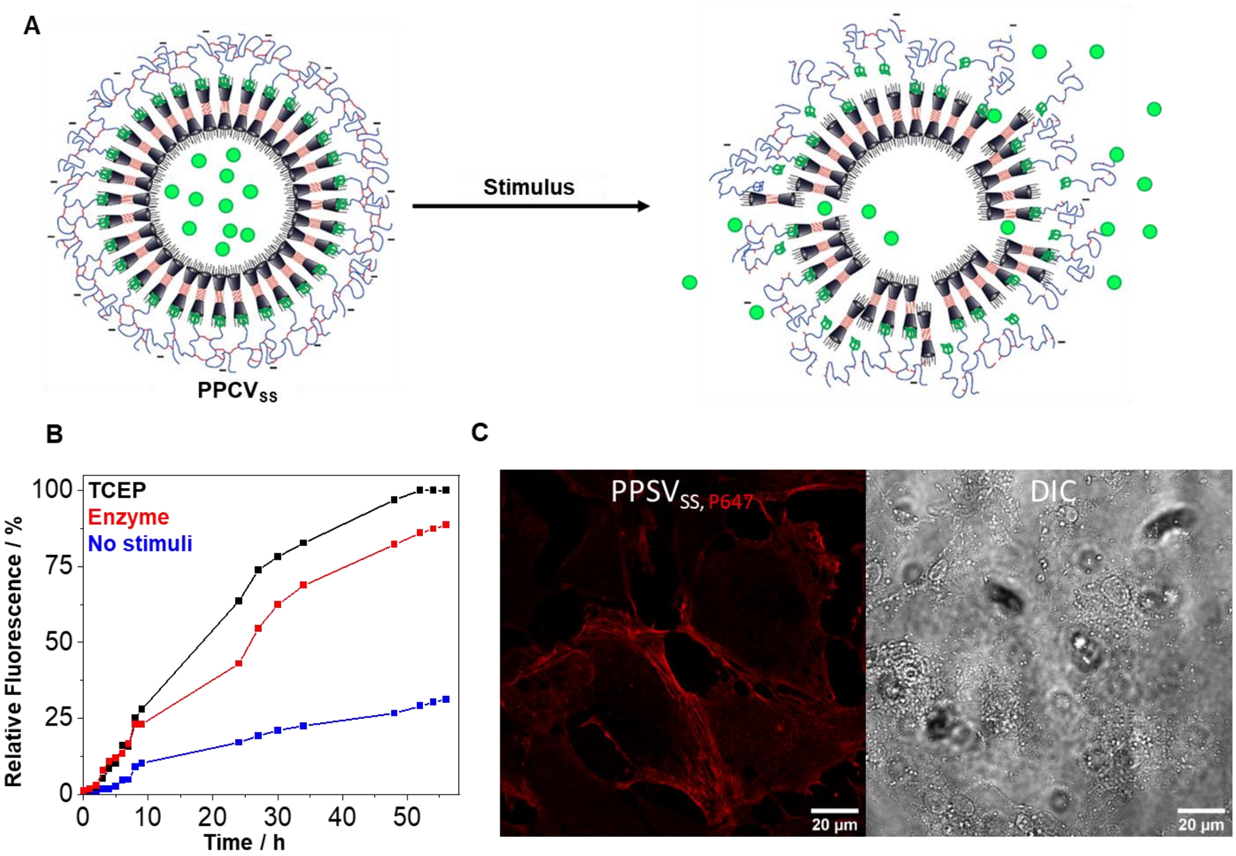

Figure S12. A. Schematic representation of the degradation of $PPSV_{SS}$ after a reductive or enzymatic stimulus. **B.** Release profile of pyranine from PPSVss in the presence and absence of TCEP and enzyme. **C.** Confocal images of HUVEC incubated with iFluor phalloidin 647-loaded $PPSV_{SS,\ P647}$ for 2 hours and imaged after 2 additional hours. The DIC channel shows morphological changes and substantial membrane blebbing due to cytotoxicity of phalloidin.

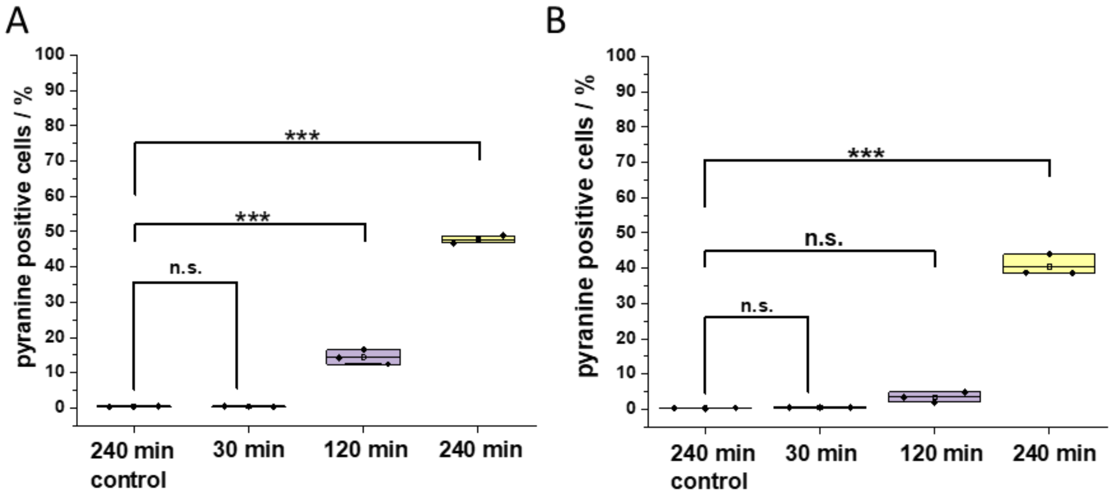

Figure S13. Quantification by flow cytometry of intracellularly released pyranine following incubation of HUVEC (**A**) or HeLa cells (**B**) with $PPSV_{SS,\ pyr}$. Cells were treated either with $PPSV_{SS}$ as a control for 240 min (representing the longest incubation time) or with $PPSV_{SS,pyr}$ for 30 min, 120 min or 240 min. Each sample was analyzed for 10,000 events in triplicates (n=3). The mean fluorescence values were obtained by subtracting the mean autofluorescence of cells from the measured mean fluorescence in the presence of released pyranine. Error box bars are standard deviations obtained from 3 independent experiments for each experimental condition. For statistical analysis, significant differences were evaluated using one-way ANOVA followed by Bonferroni correction. Differences were ranged from not significant (ns) to highly significant (***), as follows: ns, $p \geq 0.05$, * $0.01<p<0.05$; ** $0.001<p<0.01$; ***$p<0.001$.

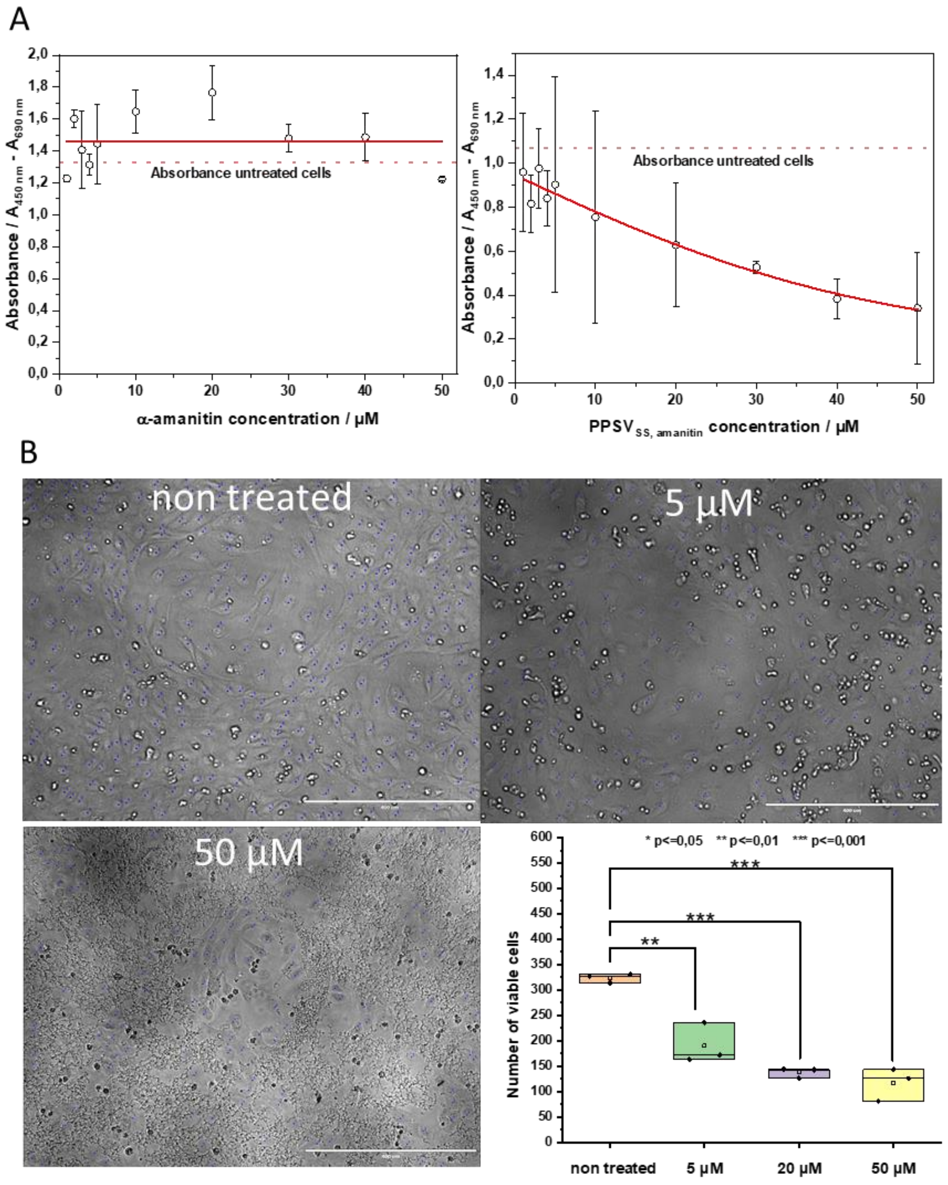

Figure S14. Effect of PPSV$_{SS,\ amanitin}$ on cell proliferation. **A.** HeLa cells were treated with PPSV$_{SS,\ amanitin}$ (right graph) or non-encapsulated α-aminitin (left graph) for 4 h at different concentrations. Cell numbers were determined by a CCK-8 assay after an additional overnight incubation. Error bars represent the mean ± SEM of three independent experiments. **B.** Phase contrast images of HUVEC incubated for 4 h with different concentration of PPSV$_{SS,\ amanitin}$, washed to remove non-internalized particles and incubated overnight at 37 °C. Cell numbers were analyzed by counting of adherent cells using Fiji Software. Scale bar 400 µm. For statistical analysis, significant differences were evaluated using one-way ANOVA followed by Bonferroni correction. Differences were ranged from not significant (ns) to highly significant (***), as follows: ns, $p \geq 0.05$, * $0.01 < p < 0.05$; ** $0.001 < p < 0.01$; ***$p < 0.001$.

Statistical analysis: Statistical analyses were performed using OriginPro 2021 (64-bit) 9.8.0.200. by using one-way ANOVA test followed by Bonferroni correction. Group sizes and definition of error bars were indicated in figure legends. Differences were ranged from not significant (ns) to highly significant (***), as follows: ns, $p \geq 0.05$, * $0.01 < p < 0.05$; ** $0.001 < p < 0.01$; ***$p < 0.001$.

References

[1] S. P. Chali, B. J. Ravoo, *Macromolecular Rapid Communications* **2020**, *41*, 2000049.
